# An Algorithmic Treatment of Causal Unit Selection †

**DOI:** 10.3390/e28050515

**Published:** 2026-05-02

**Authors:** Haiying Huang, Adnan Darwiche

**Affiliations:** Computer Science Department, University of California, Los Angeles (UCLA), Los Angeles, CA 90095, USA; darwiche@cs.ucla.edu

**Keywords:** unit selection, structural causal model, counterfactual reasoning, maximum a posteriori inference, knowledge compilation

## Abstract

The problem of optimizing a *causal* objective function emerged in recent work, where the behavior of objects needs to be expressed in terms of interventional or counterfactual probabilities. A key example is the *unit selection* problem introduced by Li and Pearl, where the goal is to find the individuals who maximize a *benefit function* that scores their characteristics (called units) using counterfactual probabilities. Previous work on unit selection focused mainly on this specific objective function and on identifying its value using bounds. We complement this line of work by developing a theory that treats unit selection as a computational problem, assuming a fully specified causal model is available and a more general class of objective functions. At the core of our treatment is a novel reduction that transforms the computation of a broad class of causal objective functions into a classical associational probability on a meta-model called the *objective model*. Based on this reduction, we propose the first exact algorithm for finding the optimal units by applying Variable Elimination (VE) on the objective model. We then characterize the complexity of causal unit selection, showing that it is ${\mathrm{NP}}^{\mathrm{PP}}$-complete, and that the runtime of VE must be exponential in the constrained treewidth of the objective model, which is larger and denser than the original input model. To address this challenge, we compile the objective model into a special class of tractable arithmetic circuits, allowing the optimal units to be computed in time linear in the circuit size. Finally, we present experiments demonstrating the substantial speedup from the circuit-based method over the VE-based method, and the speedup from the VE-based method over a baseline search method, together with a case study on a real-world ecology problem.

## 1. Introduction

A theory of causality has emerged over the last few decades based on two parallel hierarchies, an *information hierarchy* and a *reasoning hierarchy,* often called the *causal hierarchy* [1,2,3]. On the reasoning side, this theory has crystallized three levels of reasoning with increased sophistication and proximity to human reasoning: associational, interventional [1,4,5,6,7,8] and counterfactual [1,9,10,11,12], which are exemplified by the following canonical probabilities. *Associational* Pr(y|x): Probability of *y* given that *x* was observed (e.g., the probability that a patient has the flu given they have a fever). *Interventional* Pr(y|do(x)) or $\mathrm{Pr}(y_{x})$: Probability of *y* given that *x* was established by an intervention, which is different from Pr(y|x) (e.g., seeing the barometer fall tells us about the weather, but moving the barometer needle will not bring rain). *Counterfactual* $\mathrm{Pr}(y_{x}|y^{\prime },x^{\prime })$: Probability of *y* if we were to establish *x* given that neither *x* nor *y* are true (e.g., the probability that a patient who did not take a vaccine and became infected, would not have been infected had they been vaccinated). On the information side, these forms of reasoning require different levels of knowledge, encoded as associational, causal, and functional (mechanistic) models, with each class of models containing more information than the preceding one. In the framework of probabilistic graphical models [13], such knowledge is encoded by Bayesian networks [14,15], causal Bayesian networks [1,6,16] and functional Bayesian networks [17], also known as *structural causal models* (SCMs).

Reasoning about the behavior of objects under interventional or counterfactual conditions has become increasingly important across a variety of fields, including marketing, health science, recommendation systems, planning, and multi-agent decision-making. In many such settings, one would like not only to predict an object’s response to a particular action or intervention, but also to identify which objects (e.g., people, agents, regions, policies, decisions) are most desirable under a causal evaluation criterion. This has led to growing interest in optimizing *causal objective functions*, which assign a score to objects based on expressions that involve interventional or counterfactual probabilities.

Consider a motivating example in which a video-sharing platform wants to decide whether recommending a video to certain users is worthwhile. The action *X* is whether the video is shown to the user, and the target *Y* is whether the user watches it. One can use counterfactuals to categorize users into four different behavioral types: responder, always-taker, always-denier, and contrarian, based on their response with the recommendation ($Y_{x}$) and without the recommendation ($Y_{x^{\prime }}$). A responder ($y_{x},y_{x^{\prime }}^{\prime }$) would watch the video if it is recommended and would not watch otherwise. An always-taker would always watch regardless of recommendation ($y_{x},y_{x^{\prime }}$). An always-denier ($y_{x}^{\prime },y_{x^{\prime }}^{\prime }$) would always not watch regardless of the recommendation. A contrarian ($y_{x}^{\prime },y_{x^{\prime }}$) would not watch if recommended and would watch otherwise. For users observed with certain characteristics u. (e.g., age, gender, location, watch history), the expected benefit of selecting this group of users for recommendation is given by the following *benefit function* [18]:

(1) $$L\left(u\right)=\beta \mathrm{Pr}\left(y_{x},y_{x^{\prime }}^{\prime }|u\right)+\gamma \mathrm{Pr}\left(y_{x},y_{x^{\prime }}|u\right)+\theta \mathrm{Pr}\left(y_{x}^{\prime },y_{x^{\prime }}^{\prime }|u\right)+\delta \mathrm{Pr}\left(y_{x}^{\prime },y_{x^{\prime }}|u\right)$$

where Pr(·|u) denote the probability that an individual with characteristics u exhibits a given behavior type, and β, θ, γ, δ denote the benefit corresponding to each type. Note that these probabilities are *counterfactual* since they jointly consider the outcome *Y* under two conflicting scenarios. The goal is then to optimize L(u) over the domain of a fixed set of characteristic variables, U, called *unit variables* in this example, to find the specific user group that maximizes the benefit. More formally, the setup of this example can be formulated as a *Causal Unit Selection* problem [18,19]: Given a causal model *G* over variables X, a *causal objective function* L(U) mentions a special set of variables U⊆X, called *unit variables*, and can be any mathematical expression that involves associational, interventional, and counterfactual probabilities induced by model *G*. An instantiation (assignment) u of unit variables U is called a unit, and the goal is to find the unit $u^{*}$ that optimizes L(u). In contrast with classical objective functions (for example, ones used to train neural networks), it includes quantities from a higher level of the causal hierarchy: interventional or counterfactual, which gives us the ability to distinguish objects depending on their causal behavior.

Causal Unit Selection embeds two subproblems that are generally hard: *evaluation* which, involves determining the point value of the causal objective function L(u) given the available information; and *optimization*, which involves finding the unit $u^{*}$ that optimizes the score defined by L(u), assuming that the point values of the function are identifiable. Existing work on unit selection has primarily focused on the evaluation problem, under a very practical setting in which only the structure of an SCM is available together with some observational and experimental data [18,19,20,21,22]. In that setting, the data is usually not sufficient to obtain a fully specified SCM, so one cannot obtain point values of the benefit function. Furthermore, these results have largely been dedicated to the specific benefit function in Equation (1). Recent work has therefore focused on bounding the value of the benefit function while tightening these bounds as much as possible [23,24], but with less attention dedicated to optimizing benefits based on these bounds; see [22,25] for a notable exception.

In this paper, we will study unit selection from a different computational perspective and provide an algorithmic treatment of the problem that complements the previous line of work in two key ways. First, we go beyond the benefit function in Equation (1) and treat a broader class of causal objective functions. Second, we focus on the optimization problem. We assume access to a fully specified SCM in which all its parameters are known, and so we can obtain point values for any causal objective function L(u). Under this assumption, the central question then becomes: What kind of algorithms can we design to optimize such objective functions on a causal model? And how hard is it to find the optimal unit exactly, especially when the space of U and the given model is large? Particularly, we make the following contributions:

- We propose a broader class of causal objective functions that can be expressed as (linear) combination of counterfactual, interventional, or associational probabilities.
- Given a fully specified SCM, we introduce a reduction from optimizing this class of causal objective functions to the problem of optimizing a single classical associational probability, which we call *Reverse-MAP*.
- We establish the computational complexity of the unit selection problem, showing it is $NP^{PP}$-complete for this class of causal objective functions but is NP-complete when unit variables correspond to all exogenous variables in the given model.
- We propose the first exact algorithm for finding the optimal unit using Variable Elimination (VE), and characterize its complexity using constrained treewidth.
- We further propose a more efficient method based on compiling the SCM and causal objective function into a special class of Tractable Arithmetic Circuits (ACs), which allow one to compute optimal units in time linear in the circuit size.

This paper is structured as follows. Section 2 reviews Structural Causal Models and the reduction of counterfactuals into associational probabilities. Section 3 formally defines the treated causal objective functions and the unit selection problem. Section 4 presents the complexity results and the reduction to Reverse-MAP. Section 5 presents the Variable Elimination algorithm for solving unit selection exactly and its runtime complexity. Section 6 presents the more efficient method based on tractable arithmetic circuits. Finally, Section 7 presents the empirical evaluation based on random SCMs, followed by a case study based on a real-world ecological model in Section 8. Section 9 concludes the paper. This paper is a revised and expanded version of [26,27].

## 2. Background: Structural Causal Models and Counterfactual Queries

We review *structural causal models* (SCMs) in this section, since the unit selection problem is defined on these models; see [1,10,28,29] for a comprehensive exposition. We use uppercase letters (e.g., *X*) to denote variables and lowercase letters (e.g., *x*) to denote their states. We use bold uppercase letters (e.g., X) to denote sets of variables and bold lowercase letters (e.g., x) to denote their instantiations (sets of states). The states of a binary variable *X* are denoted *x* and $x^{\prime }$. We also write x∈x to mean that variable *X* has state *x* in instantiation x of variables X.

An SCM has three components. First, a directed acyclic graph (DAG) with its nodes representing variables and edges representing causal dependencies among variables. Root nodes are called *exogenous* and internal nodes are called *endogenous.* Second, a probability distribution θ(U) for each exogenous variable *U* in the model. Third, for each endogenous variable *V* with parents P, the SCM has an equation $f_{V}\left(P\right)$, called a *structural equation*, which specifies a state for *V* for each instantiation p of its parents P. Let U/V be the exogenous/endogenous variables in an SCM. The joint distribution Pr(U,V) specified by the SCM is as follows: $\mathrm{Pr}\left(u,v\right)=\prod_{u\in u}\theta \left(u\right)$ if V=v is implied by U=u and the structural equations; otherwise, Pr(u,v)=0.

SCMs are a special type of Bayesian network [14,15], which specifies a conditional probability table (CPT) for each node in the network. In particular, for node *V* with parents P, the CPT specifies the conditional distributions Pr(V|P). A structural equation can be encoded as a CPT, which contains only deterministic distributions, that is, Pr(v|p)∈{0,1} for all *v* and p. Such a CPT is said to be *functional*, and this is why SCMs are sometimes called *functional Bayesian networks.*

A Bayesian network (BN) can only be used to compute *observational* probabilities such as Pr(y|x), which is the probability of Y=y given that we *observed* X=x. An SCM can also be used to compute *interventional probabilities* such as $\mathrm{Pr}(y_{x})$, which is the probability of Y=y after we forcefully *set* X=x. The class of *causal Bayesian networks* sits between BNs and SCMs as it can be used to compute observational and interventional probabilities, but not counterfactual ones [1]. An SCM can further be used to compute *counterfactual probabilities* such as $\mathrm{Pr}(y_{x},y_{x^{\prime }}^{\prime }|e)$, which is the probability of (Y=y after setting X=x and $Y=y^{\prime }$ after setting $X=x^{\prime }$) in a situation where we observe E=e. We are particularly interested in this form of counterfactual probabilities, as it is very general and will be used as an ingredient in our objective functions. We next show how to compute such a counterfactual probability on an SCM by computing an observational probability on an auxiliary model. This will be essential for the constructions used later in the paper.

**Figure 1:**
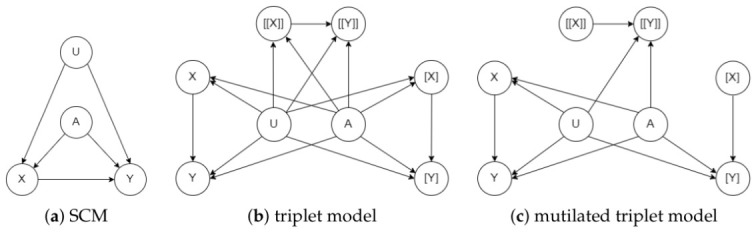
Reducing the counterfactual probability $\mathrm{Pr}(y_{x},y_{x^{\prime }}^{\prime }|x,y)$ on the model in (**a**) to an observational probability $\mathrm{Pr}(\left[y\right],\left[\left[y^{\prime }\right]\right]|\left[x\right],\left[\left[x^{\prime }\right]\right],x,y)$ on the model in (**c**).

Consider the counterfactual probability $\mathrm{Pr}(y_{x},y_{x^{\prime }}^{\prime }|x,y)$ on the SCM in Figure 1a. This query has three conflicting components: $y_{x}$, $y_{x^{\prime }}^{\prime }$ and (x,y). The first two involve conflicting actions (*x* and $x^{\prime }$). Moreover, the actions and outcomes in the first two components conflict with the observation in the third component (x,y). This is why computing counterfactual probabilities usually requires an auxiliary model that incorporates multiple worlds (real and imaginary) that all share the same causal mechanisms (exogenous variables). For the counterfactual queries we are interested in, an auxiliary model with three worlds will suffice as we discuss next.

Given an SCM *G*, its *triplet model* is another SCM constructed by having three copies $G^{1}$, $G^{2}$ and $G^{3}$ of *G* and then joining them so they share their exogenous variables; see Figure 1b. If *X* is a variable in $G^{1}$, we will use [X] to denote its copy in $G^{2}$ and [[X]] to denote its copy in $G^{3}$. A triplet model is a special case of *parallel worlds models* [30], which also include *twin models* [9]. Twin models are sufficient to evaluate counterfactual probabilities like $\mathrm{Pr}(y_{x^{\prime }}^{\prime }|x,y)$ and $\mathrm{Pr}(y_{x}^{\prime },y_{x^{\prime }})$ but not ones like $\mathrm{Pr}(y_{x},y_{x^{\prime }}^{\prime }|e)$, which we are interested in; see also [1,31]. We can now compute the counterfactual probability $\mathrm{Pr}(y_{x},y_{x^{\prime }}^{\prime }|x,y)$ on SCM *G* by operating on the triplet model as follows. First, we mutilate copies $G^{2}$ and $G^{3}$ in the triplet model by removing the edges pointing into variables [X] and [[X]] and setting [X]=x and $\left[\left[X\right]\right]=x^{\prime }$ (since we are intervening on these variables). The result is a *mutilated triplet model* shown in Figure 1c. We can then evaluate $\mathrm{Pr}(y_{x},y_{x^{\prime }}^{\prime }|x,y)$ on the SCM *G* by computing the observational probability $\mathrm{Pr}(\left[y\right],\left[\left[y^{\prime }\right]\right]|\left[x\right],\left[\left[x^{\prime }\right]\right],x,y)$ on the mutilated triplet model. Intuitively, the triplet model can be viewed as capturing three worlds $G^{1}$, $G^{2}$ and $G^{3}$. World $G^{1}$ captures the observation x, y; world $G^{2}$ captures the intervention X=x, and world $G^{3}$ captures the intervention $X=x^{\prime }$. The above treatment can be directly generalized to counterfactual queries of the form $\mathrm{Pr}(y_{x},w_{v}|e)$, where E, X, Y, V, W are sets of variables. It is precisely this class of counterfactual queries that we shall use in the rest of the paper, starting with the next section.

## 3. Causal Objective Functions and Unit Selection

A causal objective function can be any expression that involves observational, interventional or counterfactual probabilities, where the goal of the unit selection is to find objects (units) that optimize this function. However, inspired by [18], our treatment will be based on a specific class of causal objective functions, which is a linear combination of counterfactual probabilities of the form $\mathrm{Pr}({y^{i}}_{x^{i}},{w^{i}}_{v^{i}}|e^{i},u)$, where i=1,…,n. We call U the *unit variables* since our goal is to find instantiations u of these variables (i.e., units) that optimize the objective function. We note that the term “unit” is used differently in some literature, where it refers to the unit of analysis; in this paper, a unit always refers to an instantiation of variables U. Variables $X^{i}V^{i}$ represent treatments, variables $Y^{i}W^{i}$ represent outcomes, $\left(X^{i}\cup V^{i}\right)\cap \left(Y^{i}\cup W^{i}\right)=\emptyset$, and variables $E^{i}$ represent evidence. Unit variables are shared by all components of the objective function, but each component can have its own treatment, outcome, and evidence variables.

We will assume that treatment, outcome, and evidence variables are endogenous variables (roots) and unit variables cannot be descendants of treatment or outcome variables. This is consistent with the assumption in [18,19] on unit variables (also called *characteristics*). This leads us to the objective functions of the following form (the conditions we place on weights $w_{i}$ are assumed for convenience and are not restrictive for our treatment):

(2) $$L\left(u\right)=\sum_{i=1}^{n}w_{i}\cdot \mathrm{Pr}\left(y_{x^{i}}^{i},w_{v^{i}}^{i}|e^{i},u\right)\;\mathrm{where}\;\;w_{i}\geq 0$$

We can now formally define the unit selection inference problem on SCMs.

**Definition** **1** (Unit Selection)**.** *Given an SCM G, a subset* U *of its variables, and an objective function* L(u)*, such as Equation (2), the unit selection inference problem is to compute* ${\mathrm{argmax}}_{u}L\left(u\right)$*.*

The benefit function discussed in [18] has the following form:

(3) $$L\left(u\right)=\beta \mathrm{Pr}\left(y_{x},y_{x^{\prime }}^{\prime }|u\right)+\gamma \mathrm{Pr}\left(y_{x},y_{x^{\prime }}|u\right)+\theta \mathrm{Pr}\left(y_{x}^{\prime },y_{x^{\prime }}^{\prime }|u\right)+\delta \mathrm{Pr}\left(y_{x}^{\prime },y_{x^{\prime }}|u\right)$$

This class of objective functions falls as a special case of Equation (2) by setting n=4, $E^{i}=\emptyset$, $X^{i}=V^{i}=\left\{X\right\}$ and $Y^{i}=W^{i}=\left\{Y\right\}$ for i=1,…,4, where X,Y are binary variables. That is, each component *i* of the objective function uses the same single, treatment variable *X* and the same single, outcome variable *Y*. A more general form was proposed in [20] in which treatment *X* has values $x_{1},\ldots ,x_{m}$ and outcome *Y* has values $y_{1},\ldots ,y_{k}$ so the objective function can have up to $k^{m}$ components, each corresponding to a distinct response type such as $\mathrm{Pr}({y_{2}}_{x_{1}},{y_{1}}_{x_{2}},{y_{1}}_{x_{3}},{y_{3}}_{x_{4}},{y_{2}}_{x_{5}}|u)$ when k=3 and m=5. This class of objective functions is more general than Equation (2), as it allows one to express more response types, but it assumes one treatment variable and one outcome variable. The class of objective functions we consider in Equation (2) allows compound treatments and outcomes. It also allows us to seek units from a particular group. For example, if *A* and *B* are two medications (binary treatments) and *T* and *P* refer to high temperature and high blood pressure (binary outcomes), and *E* is the age group with values $e_{1},\ldots ,e_{4}$, then the objective function can include terms such as $\mathrm{Pr}({\left(t,p^{\prime }\right)}_{a,b}\;,\;{\left(t^{\prime },p^{\prime }\right)}_{a^{\prime },b}|e_{3},u)$, which is the probability that a member of the third age group would have a high temperature and normal blood pressure if administered both medications and would have normal temperature and blood pressure if administered only the second medication. Moreover, since the objective function components can have different treatment and outcome variables, one can select units based on their responses to distinct stimuli (e.g., the effect of one type of encouragement for membership renewal and the simultaneous effect of another type of encouragement on increased purchases). Going beyond the form in Equation (2), one can use causal objective functions with more general ingredients, such as the probability of a patient being a responder, given that they are not a contrarian, $\mathrm{Pr}(y_{x},y_{x^{\prime }}^{\prime }|\neg \left(y_{x}^{\prime },y_{x^{\prime }}\right))$; or the probability that a patient would not have had a stroke if they were on a diet $\left(y_{d}^{\prime }\right)$ or had exercised $\left(y_{e}^{\prime }\right)$ given that they did neither $\left(d^{\prime },e^{\prime }\right)$, i.e., $\mathrm{Pr}(y_{d}^{\prime }\vee y_{e}^{\prime }|y,d^{\prime },e^{\prime })$. Such general quantities have not been treated in the literature, but some discussions have argued for their significance and treated some special cases; e.g., [32].

## 4. Complexity of Unit Selection and Reduction to Reverse-MAP

We show next that unit selection is ${\mathrm{NP}}^{\mathrm{PP}}$-complete for the class of causal objective functions given in Equation (2). We also show that this problem is NP-complete when unit variables correspond to all exogenous variables in the SCM. For background on complexity classes that are relevant to Bayesian network inference, see [33] on the MPE decision problem being NP-complete, and [34] on the MAP decision problem being ${\mathrm{NP}}^{\mathrm{PP}}$-complete. Ref. [35] shows that computing node marginals in a Bayesian network is #P-complete. For a textbook discussion of these complexity results, see [15] (Ch. 11).

We start by providing an efficient reduction from unit selection into a variant of the well-known MAP inference problem, which we call Reverse-MAP. We then follow by studying the complexity of Reverse-MAP and the unit selection.

Recall that our goal is to find units u that maximize the value L(u) of the objective function. The first step in solving this optimization problem is to be able to evaluate the objective L(u). We next show a construction that allows us to evaluate L(u) by evaluating a single observational probability involving unit variables U but on an extended and mutilated model. This construction will serve two purposes. First, it will permit us to characterize the complexity of the unit selection when using objective functions in the form of Equation (2). Second, we will later use the construction to develop a specific algorithm for solving the unit selection problem using these objective functions.

Consider each term $\mathrm{Pr}(y_{x^{i}}^{i},w_{v^{i}}^{i}|e^{i},u)$ in Equation (2). We reviewed in Section 2 how this quantity can be reduced to a classical conditional probability on a triplet model $G^{i}$. The next step is to encode a linear combination of these conditional probabilities as a conditional probability on some model $G^{\prime }$. This is performed using the following construction.

**Definition** **2** (Objective Model)**.** *Consider an SCM G with parameters θ and the objective function L in Equation (2). The objective model* G’ *for* 〈G,L〉 *is constructed as follows with parameters* $\theta^{\prime }$*:*

*1.*  *Construct a triplet model $G^{i}$ of G for each term $\mathrm{Pr}(y_{x^{i}}^{i},w_{v^{i}}^{i}|e^{i},u)$ in L (see Section 2). Join $G^{1},\ldots ,G^{n}$ so that their unit variables U are shared. If a variable U∈U is a root, then $G^{1},\ldots ,G^{n}$ shares a single node U; if a variable U∈U is internal, then each triplet model $G^{i}$ has created three copies $U^{i}$, $\left[U^{i}\right]$, and $\left[\left[U^{i}\right]\right]$. We enforce an equality constraint among the real-world copies $U^{i}$ across $G^{1},\ldots ,G^{n}$ by adding an auxiliary node as a common child of these copies (for a discussion of this auxiliary-node method, see [15] (Ch. 5.2)). This leads to model $G^{\prime }$.*
*2.*  *Add a node H to $G^{\prime }$ as a parent of all outcome nodes $Z={\left\{\left[Y^{i}\right],\left[\left[W^{i}\right]\right]\right\}}_{i=1}^{n}$. Node H has states $h_{1},\ldots ,h_{n}$ and prior $\theta^{\prime }\left(h_{i}\right)=w_{i}$. Each node Z∈Z now has parents $P_{Z}\cup \left\{H\right\}$, where $P_{Z}$ are the parents of Z in $G^{\prime }$ before node H is added. Let $z^{i}$ be the state of Z in the corresponding instantiation $y^{i}w^{i}$ of the objective function L. The new CPT for Z is as follows:*

$P_{Z}$*H**Z*$\theta^{\prime }\left(Z|P_{Z},H\right)$p$h_{i}$$z^{i}$$\theta (z^{i}|p)$p$h_{i}$${\bar{z}}^{i}$$\theta ({\bar{z}}^{i}|p)$p${\bar{h}}_{i}$$z^{i}$1.0p${\bar{h}}_{i}$${\bar{z}}^{i}$0.0

*Here, ${\bar{z}}^{i},{\bar{h}}_{i}$ denote any states of variables Z, H that are distinct from states $z^{i},\;h_{i}$.*

We say the objective model $G^{\prime }$ has *n components,* and call *H* the *mixture variable* as it encodes a mixture of the objective function terms. The CPTs for variables $\left[Y^{i}\right],\left[\left[W^{i}\right]\right]$ in model $G^{\prime }$ reduce to their original CPTs in SCM *G* when $H=h_{i}$, and imply $\left[Y^{i}\right]=y^{i},\left[\left[W^{i}\right]\right]=w^{i}$ when $H\neq h_{i}.$ The objective L(u) in SCM *G* is a classical probability in the objective model $G^{\prime }$ (proof in Appendix A).

**Theorem** **1.** *Consider an SCM G with unit variables U. Let L be the objective function in Equation (2), and let $G^{\prime }$ be an objective model for 〈G,L〉. Let $X={\left\{\left[X^{i}\right]\right\}}_{i=1}^{n}$, $Y={\left\{\left[Y^{i}\right]\right\}}_{i=1}^{n}$, $W={\left\{\left[\left[W^{i}\right]\right]\right\}}_{i=1}^{n}$, $V={\left\{\left[\left[V^{i}\right]\right]\right\}}_{i=1}^{n}$ and $E={\left\{E^{i}\right\}}_{i=1}^{n}$. We have $L\left(u\right)={\mathrm{Pr}}^{\prime }\left(y,w|x,v,e,u\right),$ where y, w, x, v, e are the instantiations of variables Y, W, X, V, E in objective function L.*

**Figure 2:**
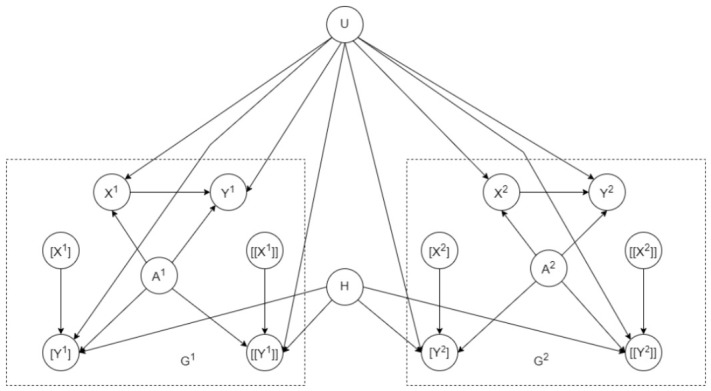
An objective model with two components for the SCM in Figure 1a.

Consider the SCM in Figure 1a and the causal objective function $L\left(u\right)=w_{1}\cdot \mathrm{Pr}\left(y_{x},y_{x^{\prime }}^{\prime }|u\right)+w_{2}\cdot \mathrm{Pr}\left(y_{x},y_{x^{\prime }}|u\right)$. Figure 2 shows a corresponding objective model $G^{\prime }$ constructed according to Definition 2. We now have $L\left(u\right)={\mathrm{Pr}}^{\prime }\left(\left[y^{1}\right],\left[\left[{y^{\prime }}^{1}\right]\right],\left[y^{2}\right],\left[\left[y^{2}\right]\right]|\left[x^{1}\right],\left[\left[{x^{\prime }}^{1}\right]\right],\left[x^{2}\right],\left[\left[{x^{\prime }}^{2}\right]\right],u\right).$

Theorem 1 suggests that we can optimize the objective function L(u) on an SCM *G* by computing the instantiation ${\mathrm{argmax}}_{u}\mathrm{Pr}\left(y,w|x,v,e,u\right)$ on an objective model $G^{\prime }$. This is similar to the classical MAP problem on model $G^{\prime }$, except that the optimized variables U appear after the conditioning operator instead of before it. This leads to our definition of the Reverse-MAP problem.

**Definition** **3** (Reverse-MAP)**.** *Consider an SCM G with distribution* Pr *and suppose* $U,\;E_{1},\;E_{2}$ *are disjoint sets of variables in G. The Reverse-MAP instantiation for variables* U *and instantiations* $e_{1},\;e_{2}$ *is defined as follows:* $\mathrm{RMAP}\left(U,e_{1},e_{2}\right)≜{\mathrm{argmax}}_{u}\mathrm{Pr}\left(e_{1}|u,e_{2}\right).$

To see the connection between Reverse-MAP and MAP, note that ${\mathrm{argmax}}_{u}\mathrm{Pr}\left(e_{1}|u,e_{2}\right)={\mathrm{argmax}}_{u}\mathrm{Pr}\left(u,e_{1},e_{2}\right)/\mathrm{Pr}\left(u,e_{2}\right)$, where ${\mathrm{argmax}}_{u}\mathrm{Pr}\left(u,e_{1},e_{2}\right)={\mathrm{argmax}}_{u}\mathrm{Pr}\left(u|e_{1},e_{2}\right)$ is the known MAP problem [14]. In general, the MAP instantiation ${\mathrm{argmax}}_{u}\mathrm{Pr}\left(u,e_{1},e_{2}\right)$ is not the Reverse-MAP instantiation since $\mathrm{Pr}(u,e_{2})$ also depends on U; see Appendix B for a concrete example that illustrates this point. We now have the following result, proven in Appendix C.

**Corollary** **1.** *There are polynomial-time reductions between the Reverse-MAP problem and the unit selection problem with objective functions in the form of Equation (2).*

We next characterize the complexity of Reverse-MAP under different conditions. Consider a decision version of the problem, D-Reverse-MAP, defined as follows.

**Definition** **4** (D-Reverse-MAP)**.** *Given an SCM with rational parameters that induces distribution* Pr*, some target variables* U*, some evidence* $e_{1},\;e_{2}$ *and a rational threshold p, the D-Reverse-MAP problems ask whether there is an instantiation* u *of* U *such that* $\mathrm{Pr}(e_{1}|u,e_{2})>p$*.*

The next theorem shows that D-Reverse-MAP is ${\mathrm{NP}}^{\mathrm{PP}}$-complete, like classical MAP [34]. Its proof can be found in Appendix D.

**Theorem** **2.** *D-Reverse-MAP is ${\mathrm{NP}}^{\mathrm{PP}}$-complete.*

We can now characterize the complexity of unit selection inference problem using Theorem 2 and Corollary 1.

**Corollary** **2.** *Unit selection is ${\mathrm{NP}}^{\mathrm{PP}}$-complete assuming the objective function in Equation (2).*

In an SCM, exogenous (root) variables represent all uncertainties in the model, and the endogenous (internal) variables are uniquely determined by exogenous variables. This property of SCMs significantly reduces the complexity of unit selection when the unit variables correspond to all SCM exogenous variables. This is implied by the following result, which is proven in Appendix E.

**Theorem** **3.** *D-Reverse-MAP is NP-complete if its target variables are all the SCM root variables.*

**Corollary** **3.** *Unit selection is NP-complete when the unit variables are all the SCM exogenous (root) variables, assuming the objective functions in Equation (2).*

## 5. Solving Unit Selection Using Variable Elimination

Section 4 provided a reduction from unit selection on an SCM to Reverse-MAP on an objective model. In Section 5.1, we provide a Variable Elimination (VE) algorithm for Reverse-MAP, which can be applied to the objective model to solve unit selection. In Section 5.2, we analyze the complexity of this method and compare it to the complexity of Reverse-MAP on the underlying SCM.

### 5.1. Reverse-MAP Using Variable Elimination

Our VE algorithm for Reverse-MAP will employ the same machinery and techniques used in the VE algorithm for classical MAP [36]. Hence, we will first review the VE algorithm for MAP using the treatment in [15] (Ch 10) and then discuss the algorithm for Reverse-MAP.

The VE algorithm is based on the notion of a *factor* f(X), which maps each instantiation x of variables X into a non-negative number f(x). VE employs a number of factor operations, including multiplying two factors (f·g), summing out a variable from a factor ($\sum_{X}f$), maximizing out a variable from a factor ($\max_{X}f$), and dividing two factors (f/g). Let *G* be an SCM and assume its variables Z are partitioned into three disjoint sets U, V, E, where U are the *target variables* and E are the *evidence variables.* Let S=Z∖U in the following discussion. We will treat the CPT of each variable *Z* in SCM *G* as a factor over *Z* and its parents P, denoted $f_{Z}\left(ZP\right)$. The SCM distribution is then $\mathrm{Pr}\left(Z\right)=\prod_{Z\in Z}f_{Z}$. We capture evidence e by creating an evidence factor $\lambda_{e}\left(E\right)$ for each e∈e with $\lambda_{e}\left(e^{\prime }\right)=1$ if $e^{\prime }=e$ and $\lambda_{e}\left(e^{\prime }\right)=0$ otherwise. The *MAP probability* is given by Equation (4). The left-hand side is a scalar (probability), while the right side is a factor over an empty set of variables, which is called a *scalar factor.* Such a factor maps only one instantiation, the empty one, to a scalar.

(4) $${\mathrm{MAP}}_{p}\left(U,e\right)=\max_{u}\mathrm{Pr}\left(u,e\right)=\max_{u}\sum_{v}\mathrm{Pr}\left(u,v,e\right)=\max_{U}\sum_{S}\prod_{Z\in Z}f_{Z}\prod_{e\in e}\lambda_{e}\left(E\right)$$

**Figure 3:**
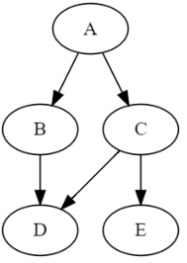
An SCM example illustrating the variable elimination procedure.

This is in contrast to the *MAP instantiation*, which is ${\mathrm{argmax}}_{u}\mathrm{Pr}\left(u,e\right)$. With some minor bookkeeping, the VE algorithm for computing the MAP probability can also return a MAP instantiation; see, e.g., [15] (Ch 10). Hence, we will focus next on computing the MAP probability. Consider the SCM in Figure 3 and suppose U={A,B} and the evidence e is {E=e}. In this case, ${\mathrm{MAP}}_{p}\left(AB,e\right)$ will be equal to

(5) $$\max_{AB}\sum_{CDE}f_{A}\left(A\right)f_{B}\left(AB\right)f_{C}\left(AC\right)f_{D}\left(BCD\right)f_{E}\left(CE\right)\lambda_{e}\left(E\right)$$

A naive evaluation of this expression multiplies all factors to yield a factor f(ABCDE) over all variables, then computes $\max_{AB}\sum_{CDE}f\left(ABCDE\right)$, leading to O(nexp(n)) complexity, where *n* is the number of model variables. The VE algorithm tries to compute this expression more efficiently with pseudocode provided in Algorithm 1 (MAP_VE). The product of factors F on Line 4 represents the joint distribution Pr(Z,e) so we first sum out variables S from F on Line 5 to compute a set of factors G whose product represents the marginal Pr(U,e). We then maximize our variables U from G on Line 6, leading to a scalar factor *p* that contains the MAP probability. The left side of Equation (4) is a scalar (probability) while the result of the right side is a factor over an empty set of variables, which is called a *scalar factor.* Such a factor maps only one instantiation, the empty one, to a scalar. Algorithm 1 eliminates variables one by one using Algorithm 2 and a total variable order $\pi =\langle \pi_{S},\pi_{U}\rangle$, known as an *elimination order.* MAP_VE requires variables U to appear last in order π since summation does not commute with maximization. An order that satisfies this constraint is known as a *U-constrained elimination order*. The complexity of MAP_VE depends on the used elimination order π. In each elimination step of Algorithm 2, we multiply all factors that mention variable π(i) to obtain factor $f_{i}$ on Line 6. The variables in $f_{i}$ are called a cluster $C_{i}$; thus, eliminating variables π(1),…,π(n) induces clusters $C_{1},\ldots ,C_{n}.$ The *width w* of elimination order π is the size of the largest cluster minus one, and the complexity of MAP_VE is O(nexp(w)).
**Algorithm 1** MAP_VE(G,U,e)**input:** SCM *G*, target variables U, evidence e**output:** scalar factor containing ${\mathrm{MAP}}_{p}\left(U,e\right)$1:**procedure** main2:      $\pi_{S}\leftarrow$ an elimination order for non-target variables3:      $\pi_{U}\leftarrow$ an elimination order for target variables U4:      $F\leftarrow \left\{f:f\;\mathrm{is}\;a\;\mathrm{CPT}\;\mathrm{of}\;\mathrm{SCM}\;G\right\}\cup \left\{\lambda_{e}:\lambda_{e}\mathrm{is}\;\mathrm{an}\;\mathrm{evidence}\;\mathrm{factor}\;\mathrm{for}\;e\in e\right\}$5:      G←eliminate($\sum ,F,\pi_{S}$)6:      p←eliminate($\max,G,\pi_{U}$)7:      **return** *p*8:**end procedure**

**Algorithm 2** Eliminating Variables using Sum or Max**input:** an operation ◯∈{∑,max}, a set of factors F, a total variable order π**output:** a set of factors1:**procedure** eliminate(◯,F,π)2:      **for** i=1 to length of order π **do**3:            $V\leftarrow i^{th}$ variable in order π4:            G← factors in F that mention variable *V*5:            $f_{i}\leftarrow \prod_{f\in G}f$6:            $f_{i}\leftarrow {◯}_{V}f_{i}$7:            replace factors G in F with factor $f_{i}$8:      **end for**9:      **return** F10:
**end procedure**


The table below depicts the trace of MAP_VE when computing the MAP probability in Equation (5) using the elimination order π=E,D,C,B,A. The trace shows that MAP_VE evaluates the following the factorized expression and that the width of order π is 2 (largest cluster has size 3):

$${\mathrm{MAP}}_{p}\left(AB,e\right)=\max_{A}f_{A}\left(A\right)\left[\max_{B}f_{B}\left(AB\right)\left[\sum_{C}f_{C}\left(AC\right)\left[\sum_{D}f_{D}\left(BCD\right)\left[\sum_{E}f_{E}\left(CE\right)\lambda_{E}\right]\right]\right]\right]$$

*i*eliminated varfactors G (Line 4, Algorithm 2)new factor $f_{i}$ (Line 6, Algorithm 2)$C_{i}$1*E*$f_{E}\left(CE\right)\;\lambda_{E}$$f_{1}=\sum_{E}f_{E}\left(CE\right)\;\lambda_{E}$CE2*D*$f_{D}\left(BCD\right)$$f_{2}=\sum_{D}f_{D}\left(BCD\right)$BCD3*C*$f_{C}\left(AC\right)\;f_{1}\left(C\right)\;f_{2}\left(BC\right)$$f_{3}=\sum_{C}f_{C}\left(AC\right)\;f_{1}\left(C\right)\;f_{2}\left(BC\right)$ABC4*B*$f_{B}\left(AB\right)\;f_{3}\left(AB\right)$$f_{4}=\max_{B}f_{B}\left(AB\right)\;f_{3}\left(AB\right)$AB5*A*$f_{A}\left(A\right)\;f_{4}\left(A\right)$$p=\max_{A}f_{A}\left(A\right)\;f_{4}\left(A\right)$A

Choosing a significant elimination order is critical for the complexity of VE. The *treewidth* of an SCM *G* is defined as the minimum width attained by any elimination order. Since MAP requires U-constrained orders, the *U-constrained treewidth* of *G* is defined as the minimum width attained by any U-constrained elimination order [34].

We are now ready to introduce our VE algorithm for Reverse-MAP. Again, we assume that the model variables Z are partitioned into disjoint sets U, V, E, where U are the target variables and S=Z∖U. But we further partition the evidence variables E into $E_{1}$ and $E_{2}$. Again, we focus on computing the Reverse-MAP probability ${\mathrm{RMAP}}_{p}\left(U,e_{1},e_{2}\right)$ instead of the instantiation:

$$\max_{u}\mathrm{Pr}\left(e_{1}|u,e_{2}\right)=\max_{u}\frac{\mathrm{Pr}(u,e_{1},e_{2})}{\mathrm{Pr}(u,e_{2})}=\max_{u}\frac{\sum_{v}\mathrm{Pr}\left(u,v,e_{1},e_{2}\right)}{\sum_{v}\mathrm{Pr}\left(u,v,e_{2}\right)}=\max_{U}\frac{\sum_{S}\prod_{Z\in Z}f_{Z}\prod_{e\in e_{1}\cup e_{2}}\lambda_{e}}{\sum_{S}\prod_{Z\in Z}f_{Z}\prod_{e\in e_{2}}\lambda_{e}}$$

Our algorithm, called RMAP_VE, runs two passes of elimination, as shown in Algorithm 3. In the first pass (Line 4), we sum out variables S under evidence $e_{1},\;e_{2}$ and in the second pass (Line 5), we sum out variables S under evidence $e_{2}$. This leads to two sets of factors $G_{1}$ and $G_{2}$, which correspond to marginal distributions $\mathrm{Pr}(U,e_{1},e_{2})$ and $\mathrm{Pr}(U,e_{2})$. Now we need to divide $\mathrm{Pr}(U,e_{1},e_{2})$ and $\mathrm{Pr}(U,e_{2})$ to compute $\mathrm{Pr}(e_{1}|U,e_{2})$. We next show that this can be performed efficiently by “dividing” $G_{1}$ and $G_{2}$, as shown on Line 8. The key idea is that if we run the two passes of elimination according to the same elimination order, then there will be a one-to-one correspondence between the factors in $G_{1}$ and $G_{2}$. Let $\left(g_{1}^{i},g_{2}^{i}\right)$ be the corresponding pairs of factors for i=1,…,k, where $k=|G_{1}\left|=\right|G_{2}|$. What we need is $\left(\prod_{i=1}^{n}g_{1}^{i}\right)/\left(\prod_{i=1}^{n}g_{2}^{i}\right)$ since this represents $\mathrm{Pr}(e_{1}|U,e_{2})$. But due to the mentioned correspondence, this equals $\prod_{i=1}^{n}g_{1}^{i}/g_{2}^{i}$. Thus, we can divide each pair of corresponding factors to obtain the set of factors G as performed on Line  8. We finally maximize our target variables U from G to obtain the Reverse-MAP probability (Line 9).
**Algorithm 3** RMAP_VE($G,U,e_{1},e_{2})$**input:** SCM *G*, target variables U, evidence $e_{1}$ and $e_{2}$**output:** scalar factor containing ${\mathrm{RMAP}}_{p}\left(U,e_{1},e_{2}\right)$1:**procedure** main2:      $\pi_{S}\leftarrow$ an elimination order for non-target variables3:      $\pi_{U}\leftarrow$ an elimination order for target variables U4:      $F_{1}\leftarrow \left\{f:f\mathrm{is}\;a\;\mathrm{CPT}\;\mathrm{of}\;\mathrm{SCM}\;G\right\}\cup \left\{\lambda_{e}:\lambda_{e}\;\mathrm{is}\;\mathrm{an}\;\mathrm{evidence}\;\mathrm{factor}\;\mathrm{for}\;e\in e_{1},e_{2}\right\}$5:      $F_{2}\leftarrow \left\{f:f\mathrm{is}\;a\;\mathrm{CPT}\;\mathrm{of}\;\mathrm{SCM}\;G\right\}\cup \left\{\lambda_{e}:\lambda_{e}\;\mathrm{is}\;\mathrm{an}\;\mathrm{evidence}\;\mathrm{factor}\;\mathrm{for}\;e\in e_{2}\right\}$6:      $G_{1}\leftarrow$eliminate($\sum ,F_{1},\pi_{S}$)7:      $G_{2}\leftarrow$eliminate($\sum ,F_{2},\pi_{S}$)8:      $G\leftarrow \{g_{1}/g_{2}:$$g_{1},g_{2}$ are corresponding factors in $G_{1},G_{2}\}$9:      p←eliminate($\max,G,\pi_{U}$)10:      **return** *p*11:**end procedure**

RMAP_VE has the same complexity as MAP_VE if both use the same elimination order. Suppose there are *k* factors in $G_{1}/G_{2}/G$ and the largest factor has size *c*. The cost of division on Line 8 is O(kexp(c)), while the cost of maximization on Line 9 is at least O(kexp(c)); thus, the cost of division is dominated by the cost of maximization. Hence, the complexity of RMAP_VE is still O(nexp(w)), where *n* is the number of variables and *w* is the width of the used U-constrained order π.

### 5.2. Bounding the Complexity of Unit Selection Using Variable Elimination

We can solve unit selection by applying RMAP_VE to an objective model of the SCM, as shown by Theorem 1. However, RMAP_VE (and MAP_VE) is expected to be more expensive on the objective model compared to the given SCM since the former is larger and denser than the latter. But how much more expensive? In particular, is RMAP_VE always tractable on the objective model when it is tractable on the underlying SCM? We consider this question next using the lens of treewidth, which is commonly used to analyze elimination algorithms. Recall also that MAP_VE and RMAP_VE have the same complexity when applied to the same SCM using the same target variables.

Our starting point is to study the treewidth of an objective model in relation to the treewidth of its underlying SCM. We will base our study on the techniques and results reported in [37], which studied the complexity of counterfactual reasoning. In particular, given an elimination order π of SCM *G*, we next show how to construct an elimination order $\pi^{\prime }$ for the objective model $G^{\prime }$ while providing a bound on the width of order $\pi^{\prime }$ in terms of the width of order π. Recall that we use [X] and [[X]] to denote the copies of the variable *X* in a triplet model, where X=[X]=[[X]] if *X* is exogenous. Moreover, if *U* is a unit variable, then $U=U^{1}=\cdots =U^{n}$ in an objective model.

**Definition** **5.** *Let G be an SCM and* $G^{\prime }$ *be a corresponding objective model with n components. If π is an elimination order for G, the corresponding elimination order* $\pi^{\prime }$ *for* $G^{\prime }$ *is obtained by replacing each non-unit variable X in π by* $X^{1},\ldots ,X^{n},\;\left[X^{1}\right],\ldots ,\left[X^{n}\right],\;\left[\left[X^{1}\right]\right],\ldots ,\left[\left[X^{n}\right]\right]$ *then appending the mixture variable H to the end of the order.*

Consider the elimination order π=A,X,Y,U for the SCM in Figure 1a. The corresponding elimination order $\pi^{\prime }$ for the objective model in Figure 2 is as follows:

$$\pi^{\prime }=A^{1},A^{2},X^{1},X^{2},\left[X^{1}\right],\left[X^{2}\right],\left[\left[X^{1}\right]\right],\left[\left[X^{2}\right]\right],Y_{1},Y_{2},\left[Y_{1}\right],\left[Y_{2}\right],\left[\left[Y_{1}\right]\right],\left[\left[Y_{2}\right]\right],U,H$$

The following bound (Theorem 5) follows from Lemma 1 and Theorem 4, which concerns *n-world models.* Given an SCM *G* and a subset U of its roots, an *n*-world model is obtained by creating *n* copies of *G* that share nodes U [37]. This notion corresponds to parallel worlds models [30] when U contains all roots of SCM *G*. An objective model with *n* components can be viewed as a 3n-world model, but with an additional mixture node *H* and some edges that originate from *H*. Lemma 1 and Theorem 5 are proven in Appendix G and Appendix H.

**Lemma** **1.** *Consider an SCM G and suppose SCM $G^{\prime }$ is obtained from G by adding a root node H as a parent of some nodes in G. If π is an elimination order for G and has width w, then $\pi^{\prime }=\langle \pi ,H\rangle$ is an elimination order for $G^{\prime }$ and has width $w^{\prime }\leq w+1$.*

**Theorem** **4** ([37])**.** *Consider an SCM G, a subset U of its roots and a corresponding n-world model $G^{\prime }$. If G has an elimination order π with width w, then there exists a corresponding elimination order $\pi^{\prime }$ of $G^{\prime }$ that has width $w^{\prime }\leq n\left(w+1\right)-1$.*

**Theorem** **5.** *Consider an SCM G and a corresponding objective model $G^{\prime }$ with n components. Let π be an elimination order for G and let $\pi^{\prime }$ be the corresponding elimination order for $G^{\prime }$. If π has width w and $\pi^{\prime }$ has width $w^{\prime }$, then $w^{\prime }\leq 3n\left(w+1\right)$.*

**Corollary** **4.** *If w is the treewidth of an SCM G and $w^{\prime }$ is the treewidth of a corresponding objective model $G^{\prime }$ with n components, then $w^{\prime }\leq 3n\left(w+1\right)$.*

As mentioned earlier, RMAP_VE and MAP_VE require a U-constrained elimination orders in which unit variables U appear last in the order. Hence, a U-constrained elimination order for an objective model must place the mixture variable *H* before U. This leads to the next definition.

**Definition** **6.** *Let G be an SCM with unit variables* U *and let* $G^{\prime }$ *be a corresponding objective model with n components. If π is a* U*-constrained elimination order for G, the corresponding* U*-constrained elimination order* $\pi^{\prime }$ *for* $G^{\prime }$ *is obtained by replacing each non-unit variable X in π by* $X^{1},\ldots ,X^{n},\;\left[X^{1}\right],\ldots ,\left[X^{n}\right],\;\left[\left[X^{1}\right]\right],\ldots ,\left[\left[X^{n}\right]\right]$ *then inserting mixture variable H just before* U*.*

Consider the U-constrained order π=A,X,Y,U for the SCM in Figure 1a. The corresponding U-constrained elimination order for the objective model in Figure 2 is

$$\pi^{\prime }=A^{1},A^{2},X^{1},X^{2},\left[X^{1}\right],\left[X^{2}\right],\left[\left[X^{1}\right]\right],\left[\left[X^{2}\right]\right],Y_{1},Y_{2},\left[Y_{1}\right],\left[Y_{2}\right],\left[\left[Y_{1}\right]\right],\left[\left[Y_{2}\right]\right],H,U$$

We now have the following bound on the U-constrained treewidth of objective models, which is somewhat unexpected when compared to the bound on treewidth. In particular, while the bound on treewidth grows linearly in the number of components in the objective model, the bound on U-constrained treewidth is independent of such a number. Moreover, the bound on U-constrained treewidth can depend on the number of unit variables, which is not the case for treewidth.

**Theorem** **6.** *Let G be an SCM with unit variables U and let $G^{\prime }$ be a corresponding objective model. If π is a U-constrained elimination order for G with width w and $\pi^{\prime }$ is the corresponding U-constrained elimination order for $G^{\prime }$ with width $w^{\prime }$, then $w^{\prime }\leq \max\left(3w+3,|U|\right)$. If the objective function in Equation (2) has one outcome variable ($Y^{i}=W^{i}=\left\{Y\right\}$ for all i), then $w^{\prime }\leq 3w+3$.*

**Corollary** **5.** *Let G be an SCM with unit variables U and let $G^{\prime }$ be a corresponding objective model. If w and $w^{\prime }$ are the U-constrained treewidths of G and $G^{\prime }$, then $w^{\prime }\leq \max\left(3w+3,|U|\right)$. Moreover, if the objective function in Equation (2) has a single outcome variable, then $w^{\prime }\leq 3w+3$.*

The above bounds can be significantly tighter depending on the objective function properties. Corollary 5 identifies one such property, which is satisfied by the benefit function in [18]; see Equation (3). Moreover, the factor 3 in these bounds is an implication of using a triplet model, which may not be necessary. Consider components $\mathrm{Pr}(y_{x^{i}}^{i},w_{v^{i}}^{i}|e^{i},u)$ in the objective function of Equation (2). If $E^{i}\;\;\;=\;\;\;\emptyset$ for all *i*, then a twin model is sufficient when building an objective model (similarly if $Y^{i}\;\;\;=\;\;\;X^{i}\;\;\;=\;\;\;\emptyset$ or $W^{i}\;\;\;=\;\;\;V^{i}\;\;\;=\;\;\;\emptyset$). The objective function in Equation (3), from [18], has $E^{i}\;\;\;=\;\;\;\emptyset$ for all *i* so it leads to the tighter bound $w^{\prime }\leq 2w+2$. More generally, if the objective function properties lead to removing the dependence on |U| in the bound of Corollary 5, then RMAP_VE on an objective model is tractable if RMAP_VE (MAP_VE) is tractable on the underlying SCM. Otherwise, the bound in Corollary 5 does not guarantee this. Recall that MAP, Reverse-MAP, and unit selection using Equation (2) are all ${\mathrm{NP}}^{\mathrm{PP}}$-complete, as shown earlier.

We note that the complexity bounds provided for MAP_VE and RMAP_VE are expressed in terms of treewidth. However, tighter bounds can be obtained using the notion of *causal treewidth* [38,39], which is no greater than treewidth and can remain bounded even when treewidth is not, by leveraging functional dependencies in SCMs [38]. This is a subject for future work.

## 6. Accelerating Unit Selection Using Tractable Arithmetic Circuits

We presented earlier an exact algorithm for solving unit selection based on Variable Elimination. The algorithm and its complexity are *structure-based*, meaning that they depend only on the structure of the causal model, and not on the specific values of its parameters (i.e., CPTs or structural equations). However, it is known that the performance of probabilistic inference algorithms can be improved significantly by exploiting the specific values or properties of the model parameters, such as 0/1 parameters, context-specific independence, functional dependencies, and parameter equality; see, e.g.,  [38,39,40,41]. These are known as local/parametric structures and are common in SCMs. We pursue an approach for exploiting local structures in this section, which compiles the objective model into a tractable Arithmetic Circuit (AC) to accelerate the search for optimal units. We first provide some background on ACs in Section 6.1. We next discuss in Section 6.2 how a special class of ACs can be used to solve MAP exactly in time linear in the AC size. We finally propose a new algorithm in Section 6.3 for solving R-MAP exactly on ACs, which can then be applied to the compiled AC from the objective model to solve unit selection.

### 6.1. Background on AC

**Figure 4:**
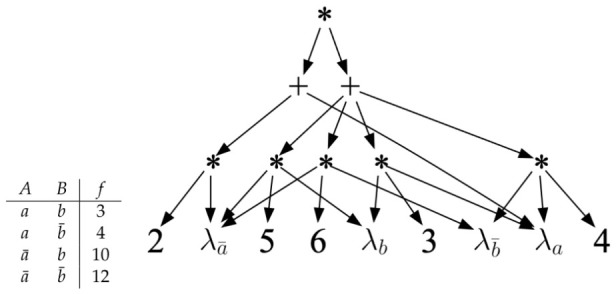
An AC that computes factor f(A,B).

An arithmetic circuit (AC) is based on a set of *discrete variables,* which define a key ingredient of the circuit: the *indicators.* For each value *x* of a variable *X*, we have an indicator $\lambda_{x}$. The AC will then have constants and indicators as its leaf nodes (inputs) with adders and multipliers as its internal nodes; see Figure 4. An AC represents a factor, which is a mapping from variable instantiations to non-negative numbers; see Figure 4. A probability distribution is a special case of a factor, so an AC can represent a distribution too, which is our focus in this work. The factor represented by an AC is obtained by evaluating the AC at complete variable instantiations. To evaluate the AC at an instantiation e, we replace each indicator $\lambda_{x}$ with 1 if the value *x* is compatible with instantiation e and with 0 otherwise [42]. We then evaluate the AC bottom-up in the standard way. The factor f(A,B) in Figure 4 has four rows, which correspond to the four instantiations of variables *A* and *B*. Evaluating the AC in this figure at each of these complete instantiations yields a value for each instantiation and therefore defines its factor. We say in this case that the AC *computes* this factor. An AC can be evaluated at a *partial* variable instantiation using the same procedure, but the value returned may not be meaningful unless the circuit satisfies certain properties. Three key such properties are decomposability, determinism, and smoothness [42]. Decomposability requires that no two indicators for the same variable may appear under two distinct children of the same ∗-node. Smoothness requires that every pair of children for a +-node mention the same set of variables (in indicators). Determinism requires at most one non-zero child for each +-node, when the circuit is evaluated under any complete variable instantiation. An AC that represents a probability distribution Pr(X) will return the marginal Pr(e) when evaluated at input e, assuming the AC is deterministic, decomposable and smooth [42]. It will also compute the MPE probability under evidence e in this case, assuming we replace +-nodes with max-nodes [43]. In fact, determinism is not needed for computing marginals, as shown initially in [44], and discussed in detail in [45]. See also [46] for a recent tutorial/survey on ACs and their properties.

### 6.2. MAP Using AC

We next discuss a special class of arithmetic circuits, decision-ACs, which can be used to solve MAP in time linear in the AC size, assuming the AC is constructed subject to specific constraints [47,48]. These constraints ensure a general condition (determinism after projection), as identified and discussed in [45], which allows MAP to be solved in linear time. We review these findings next and formalize some of the associated observations as we need them to provide a basis for our treatment of R-MAP.

**Definition** **7** (Decision-AC)**.** *Let* AC(X) *be a decomposable and smooth arithmetic circuit over variables* X*.* AC *satisfies the decision property if every +-node has the form* $n=\sum_{i}\lambda_{x_{i}}*n_{i}$*, where* $x_{i}$ *are distinct values of some variable* X∈X *and* $n_{i}$ *are circuit nodes. We say X is the decision variable of node n, denoted* dvar(n)*.*

The decision property implies determinism, so decision-ACs are decomposable, smooth, and deterministic. Decision-ACs are the numerical analog of the Boolean decision-DNNFs [49] and have been used extensively in probabilistic reasoning [42,47,48,50]. For example, the state-of-the-art ACE system [40] encodes a Bayesian Network using a CNF, compiles the CNF into a decision-DNNF using the C2D [51] compiler, and finally converts the decision-DNNF into a decision-AC. Compilation based on Variable Elimination also produces decision-ACs [15].

**Proposition** **1.** *Consider a decision-AC over variables X and let U⊆X. If the circuit satisfies the following: (1) no +-node n with dvar(n)∈U is below some +-node m with dvar(m)∉U and (2) every indicator $\lambda_{x}$ is attached to some +-node n with dvar(n)=X, then this circuit supports linear-time MAP over variables U.*

If a decision-AC satisfies Conditions (1) and (2) of Proposition 1, then we can compute ${\mathrm{MAP}}_{p}\left(U,e\right)$ exactly by traversing the AC bottom-up, while replacing every +-node *n* with a max-node if dvar(n)∈U. This was first claimed in [47] without a formal proof and was used in later works, e.g., [48,52], to solve related problems. Condition (1) was identified in [47]. Condition (2), while ensured by the ACE system [40] in practice, was never made explicit in the literature, as far as we know, and is needed for the proof of Proposition 1. Our proof of this proposition is embedded in the upcoming discussion and considers a more general condition identified in [45] which allows linear-time MAP on ACs and is satisfied by decision-ACs, as we also prove.

Let U be the MAP variables and V be all other variables. The MAP problem can be solved by first computing the marginal $\mathrm{Pr}\left(U,e\right)=\sum_{V}\mathrm{Pr}\left(X,e\right),$ which sums-out variables V (projects on variables U), and then computing the MPE $\max_{U}\mathrm{Pr}\left(U,e\right)$; see, e.g., [15]. A decomposable and smooth AC supports linear-time marginal as we can simply set all indicators of variables V to 1. If the AC is also deterministic, then it also supports linear-time MPE. However, after projecting a deterministic, decomposable, and smooth AC on variables U, the resulting AC over variables U may no longer be deterministic. If an AC remains deterministic after being projected on U, we can easily compute MAP by evaluating the circuit bottom-up while replacing every +-node that *depends on variables in U* with a max-node. This was first shown in [45] and the determinism-after-projection property was later referred to as *marginal determinism* in [53].

**Definition** **8.** *Consider a decomposable and smooth AC over variables* X *and let* U⊆X*. The AC is* **U***-deterministic* *iff the following holds: for any +-node n, if n depends on* U *(i.e.,* vars(n)∩U≠∅*), then at most one child of n can be non-zero when the AC is evaluated at any input* u *(recall that evaluating an AC at input* u *is performed by replacing every indicator* $\lambda_{x}$ *with* 0 *if x is incompatible with* u *and with* 1 *otherwise).*

If an AC is U-deterministic, then it can be used to compute the MAP probability ${\mathrm{MAP}}_{p}\left(U,e\right)$ under any evidence e by performing a bottom-up traversal, as in Equation (6):

(6) $$\mathrm{val}\left(n\right)=\left\{\begin{array}{cc} 0, & \mathrm{if}\;n\;\mathrm{is}\;\mathrm{an}\;\mathrm{indicator}\;\lambda_{x}\;\mathrm{and}\;x\;\mathrm{conflicts}\;\mathrm{with}\;e\\ 1, & \mathrm{if}\;n\;\mathrm{is}\;\mathrm{an}\;\mathrm{indicator}\;\lambda_{x}\;\mathrm{and}\;x\;\mathrm{does}\;\mathrm{not}\;\mathrm{conflict}\;\mathrm{with}\;e\\ p, & \mathrm{if}\;n\;\mathrm{is}\;a\;\mathrm{parameter}\;p\\ \prod_{c\in \mathrm{ch}(n)}\mathrm{val}\left(c\right), & \mathrm{if}\;n\;\mathrm{is}\;a\;\mathrm{product}\;\mathrm{node}\\ \max_{c\in \mathrm{ch}(n)}\mathrm{val}\left(c\right), & \mathrm{if}\;n\;\mathrm{is}\;a\;\mathrm{sum}\;\mathrm{node}\;\mathrm{and}\;\mathrm{vars}(n)\cap U\neq \emptyset \\ \sum_{c\in \mathrm{ch}(n)}\mathrm{val}\left(c\right), & \mathrm{if}\;n\;\mathrm{is}\;a\;\mathrm{sum}\;\mathrm{node}\;\mathrm{and}\;\mathrm{vars}(n)\cap U=\emptyset \end{array}\right.$$

The correctness of this procedure is established as follows. Given decomposability and smoothness, by fixing the indicators of variables V to 1, we obtain another decomposable and smooth AC that computes the projection $\mathrm{Pr}\left(U\right)=\sum_{V}\mathrm{Pr}\left(U,V\right)$. We can then reduce nodes that do not depend on U to constants (parameters). This leads to a projected AC that depends only on U, ${\mathrm{AC}}_{p}\left(U\right)$. If AC(X) is U-deterministic, then ${\mathrm{AC}}_{p}\left(U\right)$ must be deterministic, so it can compute the MPE probability $\max_{U}\mathrm{Pr}\left(U\right)$ in linear time after replacing its +-nodes with max-nodes. We finally show that decision-ACs (Definition 7) are indeed U-deterministic, which immediately implies Proposition 1, therefore showing that decision-ACs support linear-time MAP.

**Proposition** **2.** *A decision-AC is U-deterministic if it satisfies the two conditions of Proposition 1.*

The proof of this proposition can be found in Appendix J.

### 6.3. Reverse-MAP Using AC

We now consider the main question behind our proposed method for unit selection: under what conditions, and why, will an AC attain the ability to support efficient R-MAP? The answer is motivated by the following observation.

The primitive operation required by R-MAP, beyond the existing ones for classical MAP, is the ability to divide two distributions that have the same domain. That is, to obtain $\mathrm{Pr}(e_{1}|U,e_{2})$, we need to divide $\mathrm{Pr}(U,e_{1}e_{2})$ and $\mathrm{Pr}(U,e_{2})$, which is generally hard. Hence, we raise the following question: given two distributions—${\mathrm{Pr}}_{1}\left(X\right)$ and ${\mathrm{Pr}}_{2}\left(X\right)$—computed using ACs, can we efficiently obtain an AC that computes their quotient ${\mathrm{Pr}}_{3}\left(X\right)={\mathrm{Pr}}_{1}\left(X\right)/{\mathrm{Pr}}_{2}\left(X\right)$? We show that this is feasible if ${\mathrm{Pr}}_{1}\left(X\right)$ and ${\mathrm{Pr}}_{2}\left(X\right)$ are computed by two ACs with the same structure (but with different parametrizations), assuming the ACs are deterministic, decomposable, and smooth.

**Theorem** **7.** *Consider an AC that is deterministic, decomposable, and smooth under both parametrization $\Theta_{1}$ and $\Theta_{2}$. Suppose further the AC computes the distribution ${\mathrm{Pr}}_{1}\left(X\right)$ under $\Theta_{1}$ and the distribution ${\mathrm{Pr}}_{2}\left(X\right)$ under $\Theta_{2}$. Then the AC is deterministic, decomposable, and smooth and computes ${\mathrm{Pr}}_{3}\left(X\right)={\mathrm{Pr}}_{1}\left(X\right)/{\mathrm{Pr}}_{2}\left(X\right)$ under parametrization $\Theta_{3}=\left\{\theta_{1}/\theta_{2}:\theta_{1},\theta_{2}\;\mathrm{are}\;\mathrm{corresponding}\;\mathrm{parameters}\;\mathrm{in}\;\Theta_{1},\Theta_{2}\right\}$. We assume ${\mathrm{Pr}}_{2}$ has larger support than ${\mathrm{Pr}}_{1}$, i.e., ${\mathrm{Pr}}_{2}\left(x\right)=0$ only if ${\mathrm{Pr}}_{1}\left(x\right)=0$. We define 0/0=0).*

Hence, we can divide two distributions ${\mathrm{Pr}}_{1}$ and ${\mathrm{Pr}}_{2}$—induced by ACs with the same structure—by simply dividing the corresponding parameters in the ACs that lead to ${\mathrm{Pr}}_{1}$ and ${\mathrm{Pr}}_{2}$. Decomposability and smoothness are not enough, we also need determinism. The proof of Theorem 7 (in Appendix K) is based on the notion of a *complete subcircuit*, which was introduced in [43] and studied extensively in [45].

We are now ready to introduce our second, circuit-based method for solving R-MAP. Again, let U be target variables and $E_{1}$, $E_{2}$ be evidence variables. Let V=X∖U be non-target variables. Given a U-deterministic AC(X) that represents distribution Pr(X), our algorithm, called RMAP_AC, computes the R-MAP probability $\mathrm{Pr}(e_{1}\;|\;U,e_{2})$ by running a two-pass traversal on the circuit, as shown in Algorithm 4, analogous to how Variable Elimination is extended to R-MAP.

**Algorithm 4** RMAP_AC($\mathrm{AC},U,e_{1},e_{2}$)
**input:**
  AC:    U-deterministic arithmetic circuit  U:      target variables  $e_{1}$:      evidence before the conditioning operator  $e_{2}$:      evidence after the conditioning operator**output:** returns the R-MAP probability value $\max_{u}\mathrm{Pr}\left(e_{1}|u,e_{2}\right)$1:**procedure** main2:      **for** each node *n* in AC (children before parent) **do**                      *Case I: Leaf node*3:              **if** *n* is an indicator node $\lambda_{x}$ of variable *X* **then**4:                    **if** X∉U
**then**▹*n* is indicator of non-target variables5:                  $n.{\mathrm{val}}_{1},n.{\mathrm{val}}_{2}\leftarrow 1\left[x∼e_{1}e_{2}\right],1\left[x∼e_{2}\right]$6:                    **else**▹*n* is indicator of target variables7:                  n.val←18:                    **end if**9:            **else if** *n* is a parameter node *p* **then**10:                    $n.{\mathrm{val}}_{1},n.{\mathrm{val}}_{2}\leftarrow p,p$            *Case II: Internal node independent of U*11:            **else if** vars(n)∩U=∅ **then**12:                    **if** *n* is a sum node **then**▹*n* stores two sums with respect to different evidence13:                  $n.{\mathrm{val}}_{1}\leftarrow \sum_{c\in \mathrm{ch}(n)}c.{\mathrm{val}}_{1}$14:                  $n.{\mathrm{val}}_{2}\leftarrow \sum_{c\in \mathrm{ch}(n)}c.{\mathrm{val}}_{2}$15:                    **else if** *n* is a product node **then**▹*n* stores two products with respect to different evidence16:                  $n.{\mathrm{val}}_{1}\leftarrow \prod_{c\in \mathrm{ch}(n)}c.{\mathrm{val}}_{1}$17:                  $n.{\mathrm{val}}_{2}\leftarrow \prod_{c\in \mathrm{ch}(n)}c.{\mathrm{val}}_{2}$18:                    **end if**            *Case III: Internal node dependent on U*19:            **else if** vars(n)∩U≠∅ **then**▹U-dependent node stores one max or product20:                    **if** *n* is a sum node **then**21:                     $n.\mathrm{val}\leftarrow \max_{c\in \mathrm{ch}(n)}Ratio\left(c\right)$▹ replace sum with max22:                    **else if** *n* is a product node **then**23:                  $n.\mathrm{val}\leftarrow \prod_{c\in \mathrm{ch}(n)}Ratio\left(c\right)$24:                    **end if**25:            **end if**26:    **end for**27:28:        r← root node of AC29:        **return** r.val30:
**end procedure**
31:32:**function** Ratio(*c*)33:        **if** node *c* has two values **then**34:                    **return** $c.{\mathrm{val}}_{1}/c.{\mathrm{val}}_{2}$35:        **else**36:                    **return** c.val37:        **end if**38:
**end function**


To explain how Algorithm 4 works, let us partition the nodes in AC into two sets. Let ${\mathrm{AC}}_{U}$ be the set of nodes that depend on U and ${\mathrm{AC}}_{V}$ be the set of nodes independent of U, i.e., ${\mathrm{AC}}_{U}=\left\{n\in \mathrm{AC}:\mathrm{vars}\left(n\right)\cap U\neq \emptyset \right\}$ and ${\mathrm{AC}}_{V}=\left\{n\in \mathrm{AC}:\mathrm{vars}\left(n\right)\cap U=\emptyset \right\}$. Conceptually, Algorithm 4 performs two evaluations for U-independent nodes ${\mathrm{AC}}_{V}$. In the first pass, we evaluate ${\mathrm{AC}}_{V}$ bottom-up under input $e_{1}e_{2}$. This yields a projected circuit ${\mathrm{AC}}_{U}\left(U\right)$ with parametrization $\Theta_{1}$, which computes $\mathrm{Pr}(U,e_{1}e_{2})$ and is deterministic. In the second pass, we evaluate ${\mathrm{AC}}_{V}$ bottom-up under input $e_{2}$. This yields the same projected circuit ${\mathrm{AC}}_{U}\left(U\right)$ with parametrization $\Theta_{2}$, which computes $\mathrm{Pr}(U,e_{2})$ and is deterministic.

Algorithm 4 implements these two passes efficiently in a single bottom-up traversal by having every node $n\in {\mathrm{AC}}_{V}$ store two values (${\mathrm{val}}_{1}$ and ${\mathrm{val}}_{2}$). The indicators of V are set to two different inputs: ${\mathrm{val}}_{1}$ corresponding to evidence $e_{1}e_{2}$ and ${\mathrm{val}}_{2}$ corresponding to evidence $e_{2}$ (Line 5). Internal nodes in ${\mathrm{AC}}_{V}$ then evaluate the pair $\left({\mathrm{val}}_{1},{\mathrm{val}}_{2}\right)$ side by side by applying the same arithmetic operation twice: +-nodes compute two sums and ∗-nodes compute two products (Line 12–17). After this step, every node in ${\mathrm{AC}}_{V}$ has been reduced to a pair of constants, and these constants serve as parameters $\Theta_{1}$ and $\Theta_{2}$ for U-dependent nodes ${\mathrm{AC}}_{U}$. Under parametrization $\Theta_{1}$ and $\Theta_{2}$, ${\mathrm{AC}}_{U}$ computes the two marginals $\mathrm{Pr}(U,e_{1}e_{2})$ and $\mathrm{Pr}(U,e_{2})$, respectively, and is deterministic. What we need is $\mathrm{Pr}\left(e_{1}\;|\;U,e_{2}\right)=\mathrm{Pr}\left(U,e_{1}e_{2}\right)/\mathrm{Pr}\left(U,e_{2}\right)$. By Theorem 7, this can be achieved by dividing corresponding parameters in $\Theta_{1}$ and $\Theta_{2}$. As a result, ${\mathrm{AC}}_{U}\left(U\right)$ with parametrization $\Theta_{3}$ must compute $\mathrm{Pr}(e_{1}\;|\;U,e_{2})$ and remain deterministic. In Algorithm 4, this division is performed *on the fly*. Whenever a U-dependent node consumes a child *c* that still carries two values, we replace that child’s value by the ratio $c.{\mathrm{val}}_{1}/c.{\mathrm{val}}_{2}$, as shown in Line 21 and 23. We finally evaluate ${\mathrm{AC}}_{U}$ bottom-up while setting all indicates of U to 1 (Line 7) and replacing every +-node in ${\mathrm{AC}}_{U}$ with a max-node (Line 21). The R-MAP probability is returned at the root.

We now have the following result, which follows directly from the above discussion.

**Theorem** **8.** *Consider a decomposable and smooth AC(X) that represents distribution Pr(X), and let U, E1, E2 be disjoint subsets of variables X. If AC is U-deterministic, then for any evidence $e_{1}$ and $e_{2}$, running RMAP_AC (Algorithm 4) on input $\left(\mathrm{AC},U,e1,e_{2}\right)$ returns the R-MAP probability $\mathrm{Pr}(e_{1}\;|\;U,e_{2})$ in time linear in the AC size.*

## 7. Empirical Evaluation

In this section, we provide an empirical study of the two algorithms we proposed for solving unit selection problems: the VE-based RMAP_VE (Section 5.1) and the circuit-based RMAP_AC (Section 6.3). The experiments are conducted on randomly generated SCMs, and the task is to find the optimal unit for the benefit function defined in Equation (1). Our experiments will serve two purposes. First, in Section 7.2, we evaluate the asymptotic complexity of RMAP_VE by measuring the U-constrained treewidth of the constructed objective model that VE operates on, without actually running VE on large instances. This allows us to validate the theoretical bounds established in Section 5.2. We show both analytically and empirically that for a broad class of SCMs, although RMAP_VE can perform significantly better than a baseline search method, its computational cost would still exhibit an exponential growth as the SCM size and the number of unit variables increase. Second, in Section 7.3, we apply both RMAP_VE and RMAP_AC to sampled instances to find the optimal unit—the former operating on the objective model and the latter operating on a decision-AC compiled from this model—and compare their actual runtime and complexity. This allows us to demonstrate the significant speedup provided by compilation and to show that the circuit-based method is not necessarily bounded by the U-constrained treewidth in the same manner as the VE-based method.

### 7.1. Synthetic SCM Benchmarks and Their Hardness

We next describe our procedures used to generate random SCMs and unit selection problem instances. In particular, we characterize a class of problems for which the U-constrained treewidth is guaranteed to be no smaller than the number of unit variables, |U|, making them hard for VE-based methods such as RMAP_VE to solve. The random SCMs we use in this experiment resemble this class of problems, given how they are constructed.

We generate random SCMs according to the method in [37]. This method first generates a random DAG $G_{0}$ with *n* binary nodes and each node having a maximum number of parents p=6. We then convert $G_{0}$ into SCM *G* by adding a unique root parent for each internal node in $G_{0}$. The resulting DAG *G* tends to have many roots, which is meant to mimic the structure of SCMs commonly used for counterfactual reasoning. Given a random SCM structure, we randomly select different percentages ur of roots to be unit variables U. We assume the objective function given in Equation (1). This function has a single outcome variable *Y*, which we randomly choose from the SCM leaves. An ancestor (cause) of *Y* is randomly chosen as the action variable *X*. Moreover, this function has four components and, as discussed earlier, each component requires only a twin model when constructing the objective model since it does not include evidence variables.

Before we highlight the outcomes of our experiment, we provide some insight into the class of SCMs used and why they are difficult. The second step of SCM construction ensures that each internal node in the DAG has at least one unique parent that is a root by adding additional roots. Thus, if we remove these roots (and their incident edges), the remaining nodes will still be connected. When unit variables U are chosen among such roots, any two unit variables $U_{1}$ and $U_{2}$ are connected by a path $U_{1}\to \cdots \leftarrow U_{2}$ that goes through only non-unit variables (plus two endpoints $U_{1}$ and $U_{2}$). Any U-constrained elimination order must eliminate non-unit variables first, and as these variables are being eliminated, fill-in edges are gradually added along such paths in the corresponding moral graph of the DAG. By the time all non-unit variables are eliminated, there must be a direct edge between every pair of $U_{1}$ and $U_{2}$ in the moral graph, so U forms a clique of size |U|. This implies that any U-constrained elimination order has width at least |U|. This observation can be abstracted into the following lemma:

**Definition** **9.** *Consider a connected DAG G and a subset*U *of its roots. We say that* U *are external to G if the DAG remains connected after removing nodes* U *and all their incident edges.*

Markovian SCMs (each root node has a single child) satisfy the above condition.

**Lemma** **2.** *Consider a connected SCM G, a subset U of its roots, and a U-constrained elimination order π with width w. If U are external to G, then w≥|U|.*

The proof of this Lemma is provided in Appendix L. The main insight here is that MAP_VE and RMAP_VE must be exponential in the number of unit variables for this class of SCMs (and unit variables) identified by Definition 9.

### 7.2. Complexity Analysis of RMAP_VE

We provide next an experiment in which we compare the asymptotic complexities of three algorithms on the class of synthetic SCMs discussed in Section 7.1: (1) MAP_VE (Algorithm 1) for computing MAP (operates on an SCM); (2) RMAP_VE (Algorithm 3) for solving unit selection (operates on an objective model); and (3) a baseline method for solving unit selection by exhaustive search (operates on a twin model). The goal of this experiment is not to compare the actual runtime of these algorithms, but to compare the structural quantities that dominate their complexities. These quantities can be computed directly from the structure of SCM, without running VE.

- **MAP_VE**: The time complexity is O(n·exp(w)), where *n* is the number of SCM nodes and *w* is the width of a U-constrained elimination order for the SCM.
- **RMAP_VE**: The time complexity is $O(n_{1}\cdot \exp\left(w_{1}\right))$, where $n_{1}$ is the number of nodes in the objective model and $w_{1}$ is the width of a U-constrained elimination order for the objective model.
- **Baseline**: The baseline method enumerates every instantiation u and returns the one maximizing the objective L(u). Its time complexity is $O(n_{2}\cdot \exp\left(w_{2}\right))$, where $n_{2}$ is the number of nodes in the twin model used to evaluate L(u) and $w_{2}=\left|U\right|+\;$ the width of an *unconstrained* elimination order for the twin model.

Hence, we compare the complexities of these three algorithms by reporting the number of nodes *n*, $n_{1}$, $n_{2}$ and the corresponding widths *w*, $w_{1}$, $w_{2}$. These are depicted in Table 1, which also reports the number of SCM roots (*R*) and the percentage of roots used as unit variables (ur). We note that all elimination orders and width are computed using the minfill heuristic [54]. We do not prune the SCMs used by MAP_VE, the objective models used by RMAP_VE, or the twin models used by the baseline method (see [15] (Ch. 6)); thus, the choice of interventional variables does not affect our complexity analysis (no evidence variables in the objective function of Equation (1)).

We can now highlight the patterns in Table 1. The complexities of MAP_VE and RMAP_VE are relatively close, with the latter being more expensive than the former. Moreover, the gap between them narrows as the number of SCM variables (*n*) and the number of unit variables (ur) increase. Note that according to Theorem 6, $w_{1}/w\leq 2$; yet, Table 1 shows that this ratio can be significantly smaller than 2. Finally, the baseline method is significantly worse than RMAP_VE, and the gap between the two grows as the number of SCM variables (*n*) and unit variables (ur) increases.

**Figure 5:**
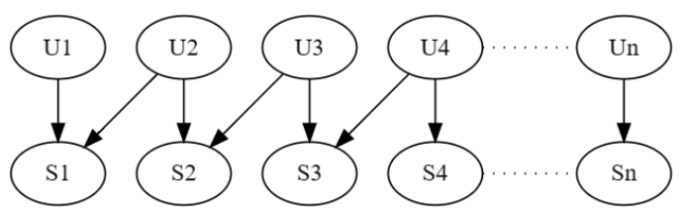
The unit variables are $U=U_{1},\ldots ,U_{n}$. The U-constrained treewidth is 3.

We close this discussion by identifying a class of problems with an unbounded number of unit variables U yet a bounded U-constrained treewidth. This class is depicted in Figure 5. The U-constrained treewidth is 3, which can be shown using the U-constrained elimination order $S_{1},\ldots ,$ $S_{n},\ldots ,U_{1},\ldots ,U_{n}.$ This is a class of problems for which unit selection using RMAP_VE is tractable even when the number of unit variables is unbounded, assuming one uses a suitable objective function (e.g., the benefit function of [18] given in Equation (3)).

### 7.3. Performance: RMAP_VE vs. RMAP_AC

We now evaluate the actual runtime and performance of the two proposed algorithms, RMAP_VE and RMAP_AC, for unit selection on the synthetic SCM benchmark. Our implementation of RMAP_AC is on top of the ACE system (http://reasoning.cs.ucla.edu/ace/ (accessed on 18 February 2026)), which we use to compile the objective model into a decision-AC, and then evaluate the AC by the two-pass traversal, as described in the previous section.

For each problem instance, we construct an objective model $G^{\prime }$ by composing triplet-models $G^{1},\ldots ,G^{4},$ one for each component in the benefit function, as discussed earlier; see [26] for more details. We then run ACE_RMAP on $G^{\prime }$ to find the optimal units and compare its results against VE_RMAP implemented in NumPy. The computation for each instance is given 10 min to complete. For each problem instance, we report the execution time of ACE_RMAP and VE_RMAP. For ACE_RMAP, it also includes the compile time of the decision-AC. We also report the size of the circuit generated by ACE (ac_size), and the total size of all factors generated by VE (ve_size), which are two comparable parameters that measure the total number of arithmetic operations required by ACE_RMAP and VE_RMAP, respectively. We also report the approximate constrained treewidth (tw) of $G^{\prime }$, which is computed using minfill heuristics [54] to find a constrained elimination order for VE_RMAP.

For each SCM size n∈{10,15,20,25,30,35,40}, we generate 25 instances and report the average statistics in Table 2. We highlight the patterns from the statistics. First, as *n* increases, the time and the number of operations of VE_RMAP grow exponentially and become impractical after n>30 (our implementation runs out of memory since NumPy does not support ndarrays with >32 dimensions). This is predicted as VE_RMAP is purely structure-based and must be exponential in the constrained treewidth (tw). Second, ACE_RMAP is much more efficient than VE_RMAP, leading to orders-of-magnitude speedups as a result of exploiting the high degree of local (parameteric) structure in the objective model (e.g., 0/1 parameters, context-specific independence, parameter equality). This enables ACE_RMAP to support very large and dense models (with tw>30) that are normally out of reach if such a parametric structure is not exploited.

## 8. Case Study

In this section, we demonstrate the utility of optimizing causal objective functions by providing case studies in ecology. We frame real-world problems in the domain as unit selection problems, using objective functions beyond the form of the benefit function (Equation (1)). We then apply the algorithms developed in Section 5 and Section 6 to solve these problems.

### 8.1. Coral Reef Regime Shifts

**Figure 6:**
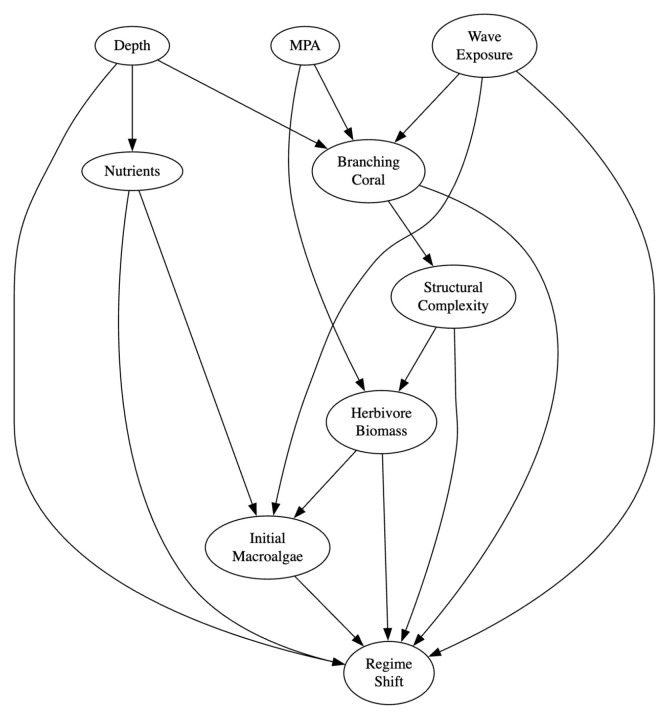
Causal DAG for coral reef regime shift from [55]. The outcome variable is Regime Shift. Treatment variables are Nutrients, Initial Macroalgae, and Herbivore Biomass. Unit variables are Depth, MPA, and Wave Exposure.

We consider a case study in climate-mediated coral reef regime shifts. The outcome variable *Y* represents whether a regime shift occurs (Y=1) or not (Y=0). In the previous study of [55], variables assumed to be relevant to regime shift were whether it was a marine protected area (MPA), herbivore biomass, wave exposure, initial macroalgae, depth, nutrients, branching coral, and structural complexity. A causal DAG, Figure 6, was proposed to capture how these variables interact. In this model, Depth (*D*), MPA (*P*), and Wave Exposure (*W*) are stable properties and can be used to identify a particular reef type, and therefore we choose them as the unit variables U. Nutrients (*N*), Initial Macroalgae (*M*), and Herbivore Biomass (*H*) describe the local environment of a reef and are variables that one can possibly intervene on, and therefore we choose them as treatment variables.

The question we are interested in is retrospective: Among reefs that did shift (Y=1), which reef types (u) would have been most likely not to shift (Y=0) had we intervened on *one of the three* treatment variables N,M,H? This question can be answered by optimizing the following causal objective function:

(7) $$L\left(u\right)=p_{1}\mathrm{Pr}\left(Y_{N=0}=0\;|\;Y=1,u\right)+p_{2}\mathrm{Pr}\left(Y_{M=0}=0\;|\;Y=1,u\right)+p_{3}\mathrm{Pr}\left(Y_{H=1}=0\;|\;Y=1,u\right)$$

where unit variables are U={Depth,MPA,WaveExposure}. The interventions N=0, M=0, H=1 correspond to setting Nutrients=low, InitialMacroalgae=low, and HerbivoreBiomass=high, respectively. The weights $p_{1},\;p_{2},\;p_{3}$ can be viewed as prior probabilities over the three possible intervention candidates, and may be chosen to be inversely related to the intervention cost. In our experiment, we initialize them as [0.3,0.1,0.6].

This objective function involves three counterfactual components and is different from the benefit function in two ways. First, each component involves a distinct treatment variable rather than a single, shared treatment variable; second, each component involves evidence that the reef did shift in reality. Despite this additional expressiveness, it falls into our considered class of causal objective functions in Equation (2).

### 8.2. Learning SCM from Data

We next learn a fully specified, discrete SCM that is consistent with the causal DAG proposed by [55] and the observational data in their Seychelles dataset, so that we can apply our unit selection algorithms on the SCM. The dataset contains 21 reef sites, and is available at https://doi.org/10.6084/m9.figshare.14981235 (accessed on 18 February 2026). Each instance provides a complete observation of all nine variables in the causal DAG shown in Figure 6. Most of these variables are continuous. Recall from Section 2 that in SCM, uncertainties are represented only through root (exogenous) variables, while interval (endogenous) variables must be deterministic functions of their parents. To obtain such an SCM from the given DAG and data, we proceed in the following four steps:

- **Construct the SCM structure**. Our starting point is the DAG in Figure 6. For each of the six internal variables *X*, we add an additional root variable $U_{X}$ (often called a background variable), which is responsible for capturing the uncertainty in $X\;|\;P_{X}$, where $P_{X}$ are the parents of *X* in the DAG. This resulted in a Markovian SCM with 15 variables in total: 9 observed variables and 6 hidden root variables.
- **Discretize the observed data**. We convert each observed variable into a binary state space using simple thresholds derived from the data. Regime Shift (*Y*) and Marine Protected Area (*P*) are already binary.
- **Learn a causal Bayesian Network (BN) over observed variables**. We learn a causal BN with the DAG structure before adding hidden roots, from the discretized data. For each variable *X* in the DAG, we learn its CPT $\mathrm{Pr}(X\;|\;P_{X})$ using a maximum likelihood (MLE) estimate with uniform smoothing. The result of this step is a fully parametrized BN over the observed variables.
- **Convert the BN to an SCM**. Finally, we convert this learned BN over observed variables into an SCM over both observed variables and hidden roots $U_{X}$. Specifically, for each internal variable *X*, we introduce a hidden root $U_{X}$, and construct $\mathrm{Pr}(U_{X})$ with a deterministic function $f_{X}\left(P_{X},U_{X}\right)$ such that after marginalizing out $U_{X}$, it produces exactly the same CPT distribution $\mathrm{Pr}(X\;|\;P_{X})$ learned in the previous step. We do this using an exact inverse–CDF transformation procedure, which is more efficient than the classical construction in [17] in terms of the number of states used by hidden variables $U_{X}$.

In general, this conversion from BN to SCM is not unique, but the resulting SCM will be consistent with the given DAG and the data over observed variables. We further acknowledge that the resulting SCM would require domain validation, but our goal here is to use this SCM mainly for an illustrative purpose and a computational evaluation.

### 8.3. Experimental Results

In this section, we apply our unit selection algorithms RMAP_VE (Section 5.1) and RMAP_AC (Section 6.3) to the coral reef SCM learned in the previous section, using the causal objective function in Equation (7). We report the optimal unit returned by our solver, the score of every unit instantiation, and illustrate the auxiliary models used to evaluate and optimize this objective function.

**Figure 7:**
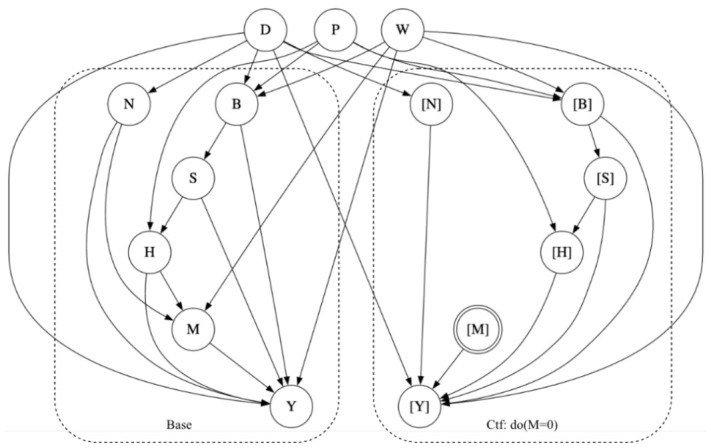
Twin model for computing $\mathrm{Pr}(Y_{M=0}=0\;|\;Y=1,u)$, the second component of the coral reef counterfactual objective. Bracketed variables denote duplicate variables in the counterfactual world.

Figure 7 shows the twin model used to compute a single counterfactual component, $\mathrm{Pr}(Y_{M=0}=0\;|\;Y=1,u)$, in the objective function. It consists of two copies of the base SCM in Figure 6: the left copy represents the real world in which the reef is observed to have shifted (Y=1), while the right copy represents the counterfactual world in which we intervene to set variable Macroalgae (M) to low. The two worlds share all root variables, including unit variables Depth (D), MPA (P), and Wave Exposure (W), as well as the hidden background variables $\left\{U_{X}\right\}$ introduced in Section 8.2 to capture uncertainty. For clarity, the hidden background variables $U_{X}$ are not explicitly shown in this graph. In the counterfactual world, the incoming edges coming to the intervened node *M* (double-circled) are removed to indicate that its value is set externally rather than observed. This construction allows the counterfactual probability $\mathrm{Pr}(Y_{M=0}=0\;|\;Y=1,u)$ to be computed as a classical associational probability on this twin model, Pr([Y]=0|[M]=0,Y=1,u).

**Figure 8:**
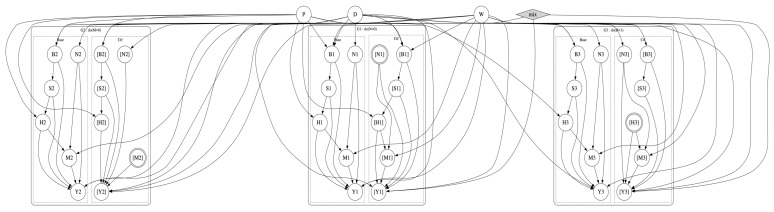
Objective model for the coral reef counterfactual objective. The model contains three counterfactual components, corresponding to $G_{1}:do\left(N=0\right)$, $G_{2}:do\left(M=0\right)$, and $G_{3}:do\left(H=1\right)$. In each component, the left dashed box represents the base world, and the right dashed box represents the counterfactual world. Shared nodes *D*, *P*, and *W* correspond to the unit variables being optimized, and the mixture node (diamond-shaped) encodes a weighted combination of these three counterfactual components. The intervened node in each component is double-circled.

Figure 8 shows the objective model used to compute the objective function in Equation (7). It is obtained by combining three twin models, corresponding to the three counterfactual components of the objective function, into a single model. In the figure, each subgraph represents one component: $\mathrm{Pr}(Y_{N=0}=0\;|\;Y=1,u)$, $\mathrm{Pr}(Y_{M=0}=0\;|\;Y=1,u)$, and $\mathrm{Pr}(Y_{H=1}=0\;|\;Y=1,u)$. The mixture node (diamond-shaped) encodes the weights of these three components, so that inference on the objective model directly computes the value of L(u). In this way, optimization of this causal objective function L(u) is reduced to a Reverse-MAP query on this objective model, $\mathrm{argmax}\mathrm{Pr}(\left[Y_{1}\right]=\;0,\left[Y_{2}\right]=\;0,\left[Y_{3}\right]=\;0|\left[N_{1}\right]=\;0,\left[M_{2}\right]=\;0,\left[H_{3}\right]=\;1,Y_{1}=\;1,Y_{2}=\;1,Y_{3}=\;1)$. For details of this objective model construction, see Section 4.

We then run our RMAP_VE and RMAP_AC solvers on the objective model in Figure 8 to solve the unit selection problem under the objective in Equation (7). The VE solver finishes in 0.151 s, while the AC solver finishes in 0.644 s. In this case, the AC solver does not yield a speedup, due to the associated overhead, because the used model is quite small: the base SCM has only 15 variables. Table 3 reports the computed scores for all eight possible units, that is, all instantiations of the unit variables U={Depth,MPA,WaveExposure} under this objective.

The optimal unit u is (1,0,1), corresponding to deep reefs outside MPAs with high wave exposure, with objective value L(u)=0.138. To interpret this quantity, it means that among reefs that did shift in reality and satisfy (Depth, MPA, WaveExposure) = (high, no, high), about 13.8% would be expected to avoid shifting if one of the three interventions were applied: reducing nutrients, reducing macroalgae, or increasing herbivore biomass. We also observe that this unit achieves the highest score on two of the three components in the objective, $\mathrm{Pr}(Y_{N=0}=0\;|\;Y=1,u)$ and $\mathrm{Pr}(Y_{H=1}=0\;|\;Y=1,u)$. Since the herbivore biomass intervention (H=1) has the largest weight in the objective, this component contributes the most to the final ranking of units.

We finally discuss several qualitative patterns in Table 3. First, reef types with Depth = 1 tend to achieve higher objective values than reef types with Depth = 0, suggesting that deeper reefs are more responsive to intervention under the learned SCM. This is consistent with the finding in a previous study [55] that deeper reefs are more resilient. Second, the effect of MPA is relatively small: for fixed Depth and Wave Exposure, changing the MPA state does not substantially improve the objective and sometimes decreases it. This is also consistent with the previous study. Third, the Wave Exposure variable interacts strongly with Depth. For shallow reefs, Wave Exposure = 1 often leads to low objective values, while for deep reefs, the combination Depth = 1 and Wave Exposure = 1 achieves the highest score overall.

## 9. Conclusions

We presented an algorithmic treatment of the problem focusing on optimization over units, which complements existing studies. We assumed a fully specified structural causal model so point values of causal objective functions can be obtained, allowing us to entertain a broader class of functions than is normally considered. Under this assumption, we introduced a reduction from optimizing this class of causal objective functions to optimizing a classical associational probability on a meta-model, called the *objective model*. We showed that the unit selection problem with this class of objective functions is ${\mathrm{NP}}^{\mathrm{PP}}$-complete, similar to the classical MAP problem, and identified an intuitive condition under which it is NP-complete. In the process, we defined a new inference problem, Reverse-MAP, which captures the essence of unit selection more than MAP does.

On the algorithmic side, we proposed two exact algorithms for solving the unit selection problem by solving Reverse-MAP on the objective model. The first algorithm is based on Variable Elimination. We characterized its complexity in terms of treewidth, while relating this complexity to that of MAP inference. The second algorithm is based on compiling the objective model into a special class of tractable arithmetic circuits, called *decision-ACs*. We showed that if a decision-AC is constructed subject to specific constraints, then the optimal unit can be computed in time linear in the AC size. We finally demonstrate the performance of our proposed algorithms on randomly generated SCMs and their practical utility by including a case study on a real-world ecology problem.

## Figures and Tables

**Table 1:** Comparing the complexities of MAP_VE for solving MAP (nexp(w)), RMAP_VE for solving unit selection ($n_{1}\exp\left(w_{1}\right)$), and the baseline method for solving unit selection ($n_{2}\exp\left(w_{2}\right)$). Each data point is an average over 25 runs.

ur			20%				40%				60%				80%				100%			
n	$n_{2}$	R	$n_{1}$	w	$w_{1}$	$w_{2}$	$n_{1}$	w	$w_{1}$	$w_{2}$	$n_{1}$	w	$w_{1}$	$w_{2}$	$n_{1}$	w	$w_{1}$	$w_{2}$	$n_{1}$	w	$w_{1}$	$w_{2}$
10	14	6	52	5.5	7.2	7.3	49	5.5	7.4	8.3	46	5.5	7.5	9.3	43	5.5	7.7	10.3	37	6.3	8.3	12.3
15	21	9	82	7.4	10.0	10.6	76	7.4	10.2	12.6	70	7.5	11.0	14.6	64	8.2	11.8	16.6	58	9.6	12.6	18.6
20	30	12	116	10.1	14.0	16.0	110	10.1	14.5	18.0	101	10.1	15.4	21.0	95	10.5	15.6	23.0	86	12.8	16.4	26.0
25	37	15	140	11.0	16.8	19.7	131	11.0	17.4	22.7	122	11.2	18.4	25.7	113	12.8	18.8	28.7	104	15.9	19.9	31.7
30	43	17	163	11.3	18.8	21.6	154	11.4	19.2	24.6	142	11.8	20.7	28.6	133	13.8	21.2	31.6	121	18.0	21.6	35.6
35	50	19	190	12.4	21.4	24.2	178	12.4	21.9	28.2	166	12.8	23.5	32.2	154	15.6	24.0	36.2	142	19.6	23.6	40.2
40	57	22	218	13.3	24.3	27.8	206	13.6	24.9	31.8	191	14.3	26.6	36.8	179	17.6	27.0	40.8	164	23.0	26.6	45.8
45	64	24	246	14.3	26.6	29.8	231	14.4	27.2	34.8	216	15.6	28.9	39.8	201	20.1	30.6	44.8	186	25.6	29.2	49.8

**Table 2:** Performance of ACE_RMAP vs. VE_RMAP on maximizing Pearl’s benefit function. Here *n* is #nodes we start with to sample SCM and $n^{\prime }$ is #nodes in the sampled SCM eventually. The Done column is the number out of 25 instances that are solved. Each data point is an average over the solved instances.

SCM			VE				ACE		
n	n^′^	|U|	Done	Time (s)	VE_Size	tw	Done	Time (s)	AC_Size
10	18.9	3.0	25	0.49	$4.92\times 10^{5}$	12.28	25	2.09	1595
15	28.4	3.4	25	1.42	$9.15\times 10^{6}$	16.88	25	3.31	4990
20	36.8	4.0	25	4.72	$1.02\times 10^{8}$	20.12	25	4.68	$1.03\times 10^{4}$
25	45.5	5.3	25	45.90	$2.42\times 10^{9}$	24.16	25	5.76	$3.90\times 10^{4}$
30	54.5	5.8	22	145.72	$8.07\times 10^{9}$	27.36	25	13.62	$1.12\times 10^{5}$
35	63.7	7.0	7	274.73	$1.05\times 10^{10}$	32.6	24	56.33	$3.57\times 10^{5}$

**Table 3:** Counterfactual scores for all unit instantiations under the coral reef counterfactual objective and its three components. Here, the unit variables are U=(Depth,MPA,WaveExposure). The optimal unit (bold) is (1,0,1), corresponding to deep reefs outside MPAs with high wave exposure.

Depth	MPA	WaveExp.	$P(Y_{N=0}=0|Y=1,u)$	$P(Y_{M=0}=0|Y=1,u)$	$P(Y_{H=1}=0|Y=1,u)$	L(u)
0	0	0	0.0000	0.1863	0.0574	0.0531
0	0	1	0.0000	0.0968	0.0000	0.0097
0	1	0	0.0000	0.2221	0.0154	0.0315
0	1	1	0.0000	0.0537	0.0000	0.0054
1	0	0	0.0131	0.0367	0.0708	0.0501
**1**	**0**	**1**	0.1396	0.0800	0.1471	**0.1381**
1	1	0	0.0188	0.0326	0.0138	0.0172
1	1	1	0.0236	0.0372	0.0530	0.0426

**Figure A1:**
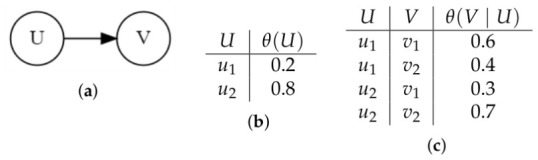
An example illustrating the difference between MAP and R-MAP (**a**) SCM; (**b**) CPT of *U*; (**c**) CPT of *V*.

## Data Availability

The original contributions presented in this study are included in the article. Further inquiries can be directed to the corresponding author.

## Appendix A. Proof of Theorem 1

The proof of this theorem requires a lemma, which requires the following definition. We will say that a set of variables Z *decomposes* a DAG if removing the outgoing edges from Z splits the DAG into at least two disconnected components.

**Lemma** **A1.**
*Consider an SCM G with distribution Pr and three disjoint sets of variables X, Y, Z. Suppose Z decomposes G into disconnected components $G_{1}$ and $G_{2}$. If $X_{1},\;Y_{1}$ are subsets of X, Y pertaining to $G_{1}$, and $X_{2},\;Y_{2}$ are subsets of X,Y pertaining to $G_{2}$, then $\mathrm{Pr}\left(y|x,z\right)=\mathrm{Pr}\left(y_{1}|x_{1},z\right)\mathrm{Pr}\left(y_{2}|x_{2},z\right)$.*

**Proof.** Since Z decomposes *G*, we have ${\mathrm{dsep}}_{G}\left(X_{1},Z,X_{2}\right)$ and ${\mathrm{dsep}}_{G}\left(X_{1}Y_{1},Z,X_{2}Y_{2}\right)$. We have:

(A1) $$\begin{array}{cc} \mathrm{Pr}(y|x,z) & =\frac{\mathrm{Pr}(y,x|z)}{\mathrm{Pr}(x|z)}=\frac{\mathrm{Pr}(y_{1},y_{2},x_{1},x_{2}|z)}{\mathrm{Pr}(x_{1},x_{2}|z)}=\frac{\mathrm{Pr}\left(y_{1},x_{1}|z\right)\mathrm{Pr}\left(y_{1},x_{1}|z\right)}{\mathrm{Pr}\left(x_{1}|z\right)\mathrm{Pr}\left(x_{2}|z\right)}\\ \; & =\left[\frac{\mathrm{Pr}(y_{1},x_{1}|z)}{\mathrm{Pr}(x_{1}|z)}\right]\left[\frac{\mathrm{Pr}(y_{2},x_{2}|z)}{\mathrm{Pr}(x_{2}|z)}\right]=\mathrm{Pr}\left(y_{1}|x_{1},z\right)\mathrm{Pr}\left(y_{2}|x_{2},z\right) \end{array}$$

Equation (A1) follows from ${\mathrm{dsep}}_{G}\left(X_{1},Z,X_{2}\right)$ and ${\mathrm{dsep}}_{G}\left(X_{1}Y_{1},Z,X_{2}Y_{2}\right)$. This concludes our proof. Although we only consider the case of two subnetworks here, it is easy to see that this lemma generalizes to an arbitrary number of subnetworks decomposed by Z. □

We are now ready to prove Theorem 1. By construction of $G^{\prime }$, U∪{H} decomposes $G^{\prime }$ into its *n* components $G^{1},G^{2},\ldots G^{n}$. We have:

 Pr′(y,w∣x,v,e,u)=∑i=1nPr′(y,w,hi∣x,v,e,u)=∑i=1nPr′(y,w∣x,v,e,u,hi)Pr′(hi∣x,v,e,u)(A2)     =∑i=1nPr′(y,w∣x,v,e,u,hi)Pr′(hi)(A3)         =∑i=1n∏j=1nPr′(yj,wj∣xj,vj,ej,u,hi)Pr′(hi)(A4)                   =∑i=1n∏j≠ipr′(yj,wj∣xj,vj,ej,u,hi)Pr′(yi,wi∣xi,vi,ei,u,hi)Pr′(hi)(A5)                   =∑i=1n∏j≠i1.0Pr′(yi,wi∣xi,vi,ei,u)wi                    =∑i=1nwiPr(yxii,wvii∣ei,u)(A6)                   =L(u)

Equation (A2) follows since the auxiliary root *H* is d-separated from X∪V∪E∪U. Equation (A3) follows from Lemma A1 since U∪{H} decomposes *G* such that all triplet models are disconnected. Equation (A4) follows from the construction of the new CPTs of $Y^{i}$ and $W^{i}$: if $H=h_{i}$, then the original CPTs of $Y^{i}$ and $W^{i}$ are preserved, and the values of $Y^{j}$ and $W^{j}$ are fixed to $y^{j}$ and $w^{j}$ for all j≠i. Equation (A5) follows from the property of the triplet network.

## Appendix B. Example for MAP and Reverse-MAP

Consider the simple model in Figure A1. We have ${\mathrm{argmax}}_{u}\mathrm{Pr}\left(u,v_{1}\right)=u_{2}$ for MAP while ${\mathrm{argmax}}_{u}\mathrm{Pr}\left(v_{1}|u\right)=u_{1}$ for R-MAP.

## Appendix C. Proof of Corollary 1

We can reduce Reverse-MAP ${\mathrm{argmax}}_{u}\mathrm{Pr}\left(e_{1}|u,e_{2}\right)$ to unit selection by choosing an objective function in the form of Equation (2) with the following settings: n=1, $w_{1}=1$, $X^{1}=\left\{\right\}$, $Y^{1}=E_{1}$ and $E^{1}=E_{2}$. This is clearly a polynomial-time reduction. We already showed a reduction from unit selection to Reverse-MAP in Theorem 1. Let |G| denote the size of SCM *G* and *n* be the number of components in the causal objective function. Here, the size of an SCM is the space needed to store the SCM structure and parameters. For example, if the SCM is represented by a functional Bayesian Network, its size is usually the total number of entries in the network CPTs. By inspecting Definition 2, we can immediately see that the time for constructing the objective model $G^{\prime }$ is O(n·|G|). Moreover, the size of objective model $G^{\prime }$ is also O(n·|G|).

## Appendix D. Proof of Theorem 2

Membership in ${\mathrm{NP}}^{\mathrm{PP}}$ is immediate. Given an instantiation u of U, it is easy to verify if u is a solution by querying the PP-oracle if $\mathrm{Pr}(e_{1}|u,e_{2})>p$ which is a problem known as D-MAR [15]. To prove hardness, we show that E-MAJSAT [56] can be reduced to D-Reverse-MAP in polynomial time, based on a slight modification of the reduction to classical MAP proposed in [34]. The E-MAJSAT problem is defined as follows. Given a Boolean formula α over Boolean variables Z=U∪V: Is there an instantiation u of U such that the majority of instantiations v of V satisfy uv⊧α (formula α holds at uv)? We show that we can answer E-MAJSAT by answering D-Reverse-MAP on an SCM $G_{\alpha }$ that simulates the formula α and that can be constructed efficiently. The SCM $G_{\alpha }$ is constructed inductively, as shown in [34] (Ref. [34] intended to construct a Bayesian network, but their construction is an SCM since all internal nodes in the network have functional CPTs), and always has a single leaf node, denoted $S_{\alpha }.$ The construction is based on three rules: (1) If α=X, then $G_{\alpha }$ has a single binary node *X* with values {0,1} and a uniform prior so $S_{\alpha }=X$; (2) If α=¬β, then $G_{\alpha }$ is constructed from $G_{\beta }$ by adding a binary node $S_{\alpha }$ as a child of $S_{\beta }\in G_{\beta }$ with structural equation $S_{\alpha }=1-S_{\beta }$; and (3) If α=β∧γ (α=β∨γ), then $G_{\alpha }$ is constructed from $G_{\beta }$ and $G_{\gamma }$ by adding a binary node $S_{\alpha }$ as a child of $S_{\beta }\in G_{\beta }$ and $S_{\gamma }\in G_{\gamma }$ with structural equation $S_{\alpha }=S_{\beta }\cdot S_{\gamma }$ ($S_{\alpha }=S_{\beta }+S_{\gamma }$). We are now ready for the last step of the proof. Given a Boolean formula α over variables Z=U∪V, and given its SCM $G_{\alpha }$ that has distribution Pr, we next show that there is an instantiation u such that $\mathrm{Pr}(S_{\alpha }=1|u)>1/2$ (D-Reverse-MAP query) iff there is an instantiation u such that the majority of instantiations v of V satisfy uv⊧α (E-MAJSAT query). Let |Z|=n and $s_{\alpha }$ denote $S_{\alpha }=1$. By construction of $G_{\alpha }$ [34], we have $\mathrm{Pr}\left(z\right)=1/2^{n}$ for all instantiations z; $\mathrm{Pr}(s_{\alpha }|z)=1$ if z⊧α and $\mathrm{Pr}(s_{\alpha }|z)=0$ otherwise. Then $\mathrm{Pr}\left(z,s_{\alpha }\right)=\mathrm{Pr}\left(s_{\alpha }|z\right)\mathrm{Pr}\left(z\right)=1/2^{n}$ if z⊧α and $\mathrm{Pr}(z,s_{\alpha })=0$ otherwise. We finally have:

$$\mathrm{Pr}\left(s_{\alpha }|u\right)=\frac{\mathrm{Pr}(u,s_{\alpha })}{\mathrm{Pr}(u)}=\frac{\sum_{v}\mathrm{Pr}\left(u,v,s_{\alpha }\right)}{\sum_{v}\mathrm{Pr}\left(u,v\right)}=\frac{\sum_{v:uv⊧\alpha }\left(1/2^{n}\right)}{\sum_{v}\left(1/2^{n}\right)}=\frac{\mathrm{card}(\{v\in V:uv⊧\alpha \})}{\mathrm{card}(V)}$$

Now that we have shown membership and hardness, D-Reverse-MAP is ${\mathrm{NP}}^{\mathrm{PP}}$-complete.

## Appendix E. Proof of Theorem 3

Recall Definition 4 of D-Reverse-MAP: Is there an instantiation u such that $\mathrm{Pr}(e_{1}|u,e_{2})$ > *p*? Membership in NP is immediate. Since U is the set of exogenous variables, evidence variables E are functionally determined by U. Hence, it is easy to check whether an instantiation u is a solution by first computing the instantiation e of E implied by u (using structural equations) and then checking whether e is consistent with $e_{1},e_{2}$. If the answer is yes, then $\mathrm{Pr}(e_{1}|u,e_{2})=1$, otherwise $\mathrm{Pr}(u,e_{2})=0$ or $\mathrm{Pr}(e_{1}|u,e_{2})=0$. To show hardness, we show that SAT can be reduced to D-Reverse-MAP under the conditions stated in the theorem. Given a Boolean formula α over variables U, we construct an SCM $G_{\alpha }$ as in the proof of Theorem 2. SCM $G_{\alpha }$ has a single leaf node $S_{\alpha }$ and its root nodes are U. By construction of $G_{\alpha }$, we have $\mathrm{Pr}(S_{\alpha }=1|u)=1$ if u satisfies α and $\mathrm{Pr}(S_{\alpha }=1|u)=0$ otherwise. By choosing p=0, $e_{1}=\left\{S_{\alpha }=1\right\}$ and $e_{2}=\emptyset$, the D-Reverse-MAP query (is there u such that $\mathrm{Pr}(S_{\alpha }=1|u)>0$) answers yes iff there is an instantiation u that satisfies the formula α (SAT query). This concludes our proof.

## Appendix F. Review of Elimination Concepts

We review here the standard notions of elimination process, clusters, and moral graphs, which we use in some of the upcoming proofs; see [15] (Ch 9) for a detailed treatment.

The *moral graph* of an SCM *G* is obtained from *G* by adding an undirected edge between every pair of common parents and then undirecting all edges. *Eliminating* a variable *X* from a graph *G* is performed by connecting every pair of neighbors for *X* in *G*, and then removing node *X* from *G*. Eliminating variables from an SCM *G* is performed by eliminating variables from its moral graph $G^{\prime }$. Eliminating variables from a moral graph $G^{\prime }$ using variable order π induces a *graph sequence* $G^{\prime }=G_{1},\ldots ,G_{n}$ where graph $G_{i+1}$ is obtained by eliminating variable π(i) from $G_{i}$. We use $G_{i}\left(X\right)$ to denote *X* and its neighbors in graph $G_{i}$. We also use C(X) to denote the *cluster* of variable *X*, which is *X* and its neighbors just before eliminating *X*. If X=π(i), then $C\left(X\right)=G_{i}\left(X\right)$. We also use $C_{i}$ to denote the cluster for *X*, C(X), in this case.

## Appendix G. Proof of Lemma 1

This proof uses the elimination concepts and notations reviewed in Appendix F.

Let $G_{m}^{\prime }$ be the moral graph of $G^{\prime }$ and $C_{i}^{\prime }$ be the cluster induced by eliminating variable $X_{i}$ from $G^{\prime }$. Let *n* be the number of variables in *G*. To prove Lemma 1, it suffices to prove the following statement: $C_{i}^{\prime }\subseteq C_{i}\cup \left\{H\right\}$ for i=1,…,n, which we prove next by induction.

Let neigh(X) denote the neighbors of *X* in $G_{m}$ and let ${\mathrm{neigh}}^{\prime }\left(X\right)$ denote the neighbors of *X* in $G_{m}^{\prime }$. Let Z denote the children of *H* in $G^{\prime }$. For each Z∈Z, let $P_{Z}$ denote the parents of *Z* in *G*. First, we show the statement holds when i=1. When creating $G_{m}^{\prime }$ from $G^{\prime }$, the introduction of node *H* would cause two classes of edges that do not exist in $G_{m}$ to be added to $G_{m}^{\prime }$: (1) (Z,H) for Z∈Z; (2) (Y,H) if *Y* is a parent of some node Z∈Z, that is *Y* and *H* are common parents of some node *Z*. This means that before the elimination starts, for any node *X*, if $X\in Z\cup_{Z\in Z}P_{Z}$, we have ${\mathrm{neigh}}^{\prime }\left(X\right)=\mathrm{neigh}\left(X\right)\cup \left\{H\right\}$; otherwise ${\mathrm{neigh}}^{\prime }\left(X\right)=\mathrm{neigh}\left(X\right)$. Hence, $C_{1}^{\prime }\subseteq \left\{C_{1}\right\}\cup \left\{H\right\}$. Consider now the elimination of $X_{i+1}$ assume that the statement holds for 1,2,…,i. We observe that if $C_{j}^{\prime }\subseteq C_{j}\cup \left\{H\right\}$, then the elimination of $X_{j}$ would cause only one type of additional edges to be added to $G_{m}^{\prime }$, that is (Y,H) for $Y\in \mathrm{neigh}(X_{j})$. This is because the elimination of $X_{j}$ will form a clique among $\mathrm{neigh}\left(X_{j}\right)\cup \left\{H\right\}$ in $G_{m}^{\prime }$, but $\mathrm{neigh}(X_{j})$ already forms a clique after $X_{j}$ is eliminated from $G_{m}$. This implies that eliminating $X_{j}$ (j≤i) will never cause any additional edge to be added among any two nodes that are both not *H* (in other words, all additional edges added are incident on *H*). Thus, before we eliminate $X_{i+1}$, we have ${\mathrm{neigh}}^{\prime }\left(X_{i+1}\right)\subseteq \mathrm{neigh}\left(X_{i+1}\right)\cup \left\{H\right\}$ and this implies $C_{i+1}^{\prime }\cap C_{i+1}\cup \left\{H\right\}$ which concludes the proof.

## Appendix H. Proof of Theorem 5

An *N*-world model is obtained by creating *N* copies of a directed acyclic graph (DAG) while joining them so a *subset* of their roots are shared [37]. Hence, an objective model, as in Definition 2, corresponds to an *N*-world model except for the addition of mixture node *H* and its outgoing edges. Before adding the mixture node *H*, an objective model with *n* components corresponds to a 3n-world model so its treewidth is ≤3n(w+1)−1 by Theorem 4. After adding node *H*, its treewidth is ≤3n(w+1) by Lemma 1.

## Appendix I. Proof of Theorem 6

This proof uses the elimination concepts and notations reviewed in Appendix F.

For a node *X* in an SCM *G*, we use ${\left[X\right]}^{k}$ to denote its $k^{th}$ duplicate in an *n*-world model of *G*. If node *X* is shared between all *n* worlds, then ${\left[X\right]}^{k}=X$ for all *k*. For a set of variables X, we use ${\left[X\right]}^{k}$ to denote $\left\{{\left[X\right]}^{k}:X\in X\right\}$.

Let *G* be an SCM, U be a subset of its roots (unit variables), and let $G^{\prime }$ be a corresponding objective model with *n* components. Our proof is based on constructing an augmented objective model $G^{\prime \prime }$ by adding edges to $G^{\prime }$ and then showing that the bounds of Theorem 6 hold for $G^{\prime \prime }$. Our proof is based on Lemmas A2 and A4, which we formally state and prove later:

– Lemma A2 complements Theorem 4 by showing that any U-constrained elimination order for an SCM can be converted into a U-constrained elimination order for a corresponding *n*-world model while preserving the width of the order.
– Lemma A4 concerns the augmentation of an SCM by a root node *H* and some edges that originate from *H*. In particular, given a U-constrained elimination order of width *w* for the SCM, the lemma shows how to construct a U-constrained elimination order for its augmentation with width ≤max(w+1,|U|).

We start by showing how to construct the augmented objective model $G^{\prime \prime }$ from $G^{\prime }$. Let *H* be the mixture node of $G^{\prime }$ and $Z={\left\{Y^{i},W^{i}\right\}}_{i=1}^{n}$ be the set of all outcome variables in the objective function of Equation (2). We obtain $G^{\prime \prime }$ by adding to $G^{\prime }$ an edge H→Z for each Z∈Z if such an edge does not already exist in $G^{\prime }$. The edges of $G^{\prime \prime }$ are a superset of the edges of $G^{\prime }$, so it suffices to show that the bounds of Theorem 6 hold for $G^{\prime \prime }$. We will next use $G^{t}$ to denote a triplet (3-world) model of *G*. We will also use $G^{b}$ to denote the augmentation of $G^{t}$ with mixture node *H* and edges H→Z for Z∈Z. Note that the augmented objective model $G^{\prime \prime }$ corresponds to *n* copies of $G^{b}$ that share root nodes U∪{H}. Hence, $G^{\prime \prime }$ is an *n*-world model of $G^{b}$.

Let π be a U-constrained elimination order for *G* with width *w*. Since $G^{t}$ is a triplet (3-world) model of *G*, Theorem 4 tells us that there exists an elimination order $\pi^{t}$ of $G^{t}$ with width $w^{t}$ such that $w^{t}\leq 3w+2$ (order $\pi^{t}$ will also be U-constrained). Recall that $G^{b}$ is obtained from $G^{t}$ by adding a root node *H* and some edges that emanate from *H*. By Lemma A4, there exists an elimination order $\pi^{b}$ for $G^{b}$ with width $w^{b}$ such that $w^{b}\leq \max(w^{t}+1,|U|)=\max\left(3w+3,|U|\right)$. Moreover, if the objective function has a single outcome variable *Y*, then *H* has a single child *Y* in $G^{b}$, so, also by Lemma A4, we have $w^{b}\leq 3w+3$. Since $G^{\prime \prime }$ is an *n*-world model of $G^{b}$ based on roots U∪{H} of $G^{b}$, we have $w^{\prime \prime }=w^{b}$ by Lemma A2. In summary, we have $w^{\prime }\leq w^{\prime \prime }\leq \max\left(3w+3,|U|\right)$. If the objective function has a single outcome variable, we have $w^{\prime }\leq 3w+3$. This concludes the proof of Theorem 6.

We will next formally state and prove Lemmas A2 and A4, which we used in the above proof.

**Lemma** **A2.**
*Consider an SCM G, a subset U of its roots, and a corresponding n-world model $G^{\prime }$ for G that shares U. If w is the width of a U-constrained elimination order π for G, and $w^{\prime }$ is the width of the corresponding U-constrained elimination order $\pi^{\prime }$ for $G^{\prime }$, then $w=w^{\prime }$.*

Given an elimination order π for an SCM *G*, we can convert it into a corresponding elimination order $\pi^{\prime }$ for its *n*-world model $G^{\prime }$ (referenced in the above lemma) by replacing each variable X∉U in π with its duplicates ${\left[X\right]}^{1},{\left[X\right]}^{2},\ldots ,{\left[X\right]}^{n}$, as in Definition 2 in [37]. If π is U-constrained, then $\pi^{\prime }$ will also be U-constrained. Moreover, we define a graph sequence for the *n*-world model $G_{1}^{\prime },G_{2}^{\prime },\ldots ,G_{n}^{\prime }$ where $G_{1}^{\prime }$ is the moral graph of $G^{\prime }$, and $G_{i+1}^{\prime }$ is obtained by eliminating all duplicates of variable π(i), i.e., ${\left[\pi (i)\right]}^{1},{\left[\pi (i)\right]}^{2},\ldots ,{\left[\pi (i)\right]}^{n}$, from $G_{i}^{\prime }$.

**Proof****.** Suppose we eliminate variables from $G/G^{\prime }$ using orders $\pi /\pi^{\prime }$. We claim that at every elimination step *i*, the following properties hold:

(A) For each node X∉U, $G_{i}^{\prime }\left({\left[X\right]}^{k}\right)={\left[G_{i}\left(X\right)\right]}^{k}$
(B) For each node U∈U, $G_{i}^{\prime }\left(U\right)=\cup_{k=1}^{n}{\left[G_{i}\left(U\right)\right]}^{k}$

We next show that properties (A), (B) imply $w^{\prime }=w$ and then prove these properties. Let Y=π(i). If Y∉U, then when its duplicate ${\left[Y\right]}^{k}$ is eliminated from $G^{\prime }$, we have $C^{\prime }\left({\left[Y\right]}^{k}\right)={\left[C(Y)\right]}^{k}$. If Y∈U, then when *Y* is eliminated from $G^{\prime }$, we have $C^{\prime }\left(Y\right)=\cup_{k=1}^{n}{\left[G_{i}\left(Y\right)\right]}^{k}=G_{i}\left(Y\right)=C\left(Y\right)$ since all non-shared nodes have been eliminated before *Y*, i.e., ${\left[G_{i}\left(Y\right)\right]}^{k}=G_{i}\left(Y\right)$. This means that the cluster induced by eliminating a variable from $G^{\prime }$ always has the same size as the cluster induced by eliminating the corresponding variable from *G*, which implies $w^{\prime }=w$. We next prove properties (A), (B) by induction. By definition of an *n*-world model, these properties hold initially for $G_{1}^{\prime }$. Suppose they hold for $G_{i}^{\prime }$ and consider $G_{i+1}^{\prime }$. Let Y=π(i). Then $G_{i+1}^{\prime }$ is the result of eliminating nodes ${\left[Y\right]}^{1},\ldots ,{\left[Y\right]}^{n}$ from $G_{i}^{\prime }$. We consider two cases. **Case:** Y∉U. Consider each node *Z* in $G_{i}^{\prime }$. If *Z* is not a neighbor of ${\left[Y\right]}^{1},\ldots ,{\left[Y\right]}^{n}$ in $G_{i}^{\prime }$, then $G_{i+1}^{\prime }\left(Z\right)$ will not be affected by the elimination of ${\left[Y\right]}^{1},\ldots ,{\left[Y\right]}^{n}$ and the properties hold by the induction hypothesis. Otherwise, node *Z* falls into two cases: (A) a duplicate ${\left[X\right]}^{k}$ of a node X∉U, (B) a shared node U∈U.

(A) by the induction hypothesis, neighbors of ${\left[X\right]}^{k}$ in $G_{i}^{\prime }$ must belong to the *k*-th world, so ${\left[X\right]}^{k}$ can only be a neighbor of the *k*-th duplicate ${\left[Y\right]}^{k}$. This means that $G_{i+1}^{\prime }\left({\left[X\right]}^{k}\right)$ can only be affected by the elimination of ${\left[Y\right]}^{k}$. By the definition of Variable Elimination, we have:
  $$\begin{array}{ccc} G_{i+1}^{\prime }\left({\left[X\right]}^{k}\right) & =G_{i}^{\prime }\left({\left[X\right]}^{k}\right)\cup G_{i}^{\prime }\left({\left[Y\right]}^{k}\right)\setminus \left\{{\left[Y\right]}^{k}\right\}\\ \; & ={\left[G_{i}\left(X\right)\right]}^{k}\cup {\left[G_{i}\left(Y\right)\right]}^{k}\setminus \left\{{\left[Y\right]}^{k}\right\} & \mathrm{by}\;\mathrm{the}\;\mathrm{induction}\;\mathrm{hypothesis}\\ \; & ={\left[G_{i}\left(X\right)\cup G_{i}\left(Y\right)\setminus \left\{Y\right\}\right]}^{k}\\ \; & ={\left[G_{i+1}\left(X\right)\right]}^{k} & \mathrm{by}\;\mathrm{definition}\;\mathrm{of}\;\mathrm{Variable}\;\mathrm{Elimination} \end{array}$$
  This proves property (A).
(B) by the induction hypothesis, *U* must be a neighbor of all duplicates ${\left[Y\right]}^{1},\ldots ,{\left[Y\right]}^{n}$. We have:
  $$\begin{array}{ccc} G_{i+1}^{\prime }\left(U\right) & =G_{i}^{\prime }\left(U\right)\overset{n}{\underset{k=1}{\cup }}G_{i}^{\prime }\left({\left[Y\right]}^{k}\right)\setminus {\left\{{\left[Y\right]}^{k}\right\}}_{k=1}^{n}\\ \; & =\left(\overset{n}{\underset{k=1}{\cup }}{\left[G_{i}\left(U\right)\right]}^{k}\right)\left(\overset{n}{\underset{k=1}{\cup }}{\left[G_{i}\left(Y\right)\right]}^{k}\right)\setminus {\left\{{\left[Y\right]}^{k}\right\}}_{k=1}^{n} & \mathrm{by}\;\mathrm{the}\;\mathrm{induction}\;\mathrm{hypothesis}\\ \; & =\overset{n}{\underset{k=1}{\cup }}{\left[G_{i}\left(U\right)\right]}^{k}\cup {\left[G_{i}\left(Y\right)\right]}^{k}\setminus \left\{{\left[Y\right]}^{k}\right\}\\ \; & =\overset{n}{\underset{k=1}{\cup }}{\left[G_{i}\left(U\right)\cup G_{i}\left(Y\right)\setminus \left\{Y\right\}\right]}^{k}\\ \; & =\overset{n}{\underset{k=1}{\cup }}{\left[G_{i+1}\left(U\right)\right]}^{k} & \mathrm{by}\;\mathrm{definition}\;\mathrm{of}\;\mathrm{Variable}\;\mathrm{Elimination} \end{array}$$
  This proves property (B).

**Case:** Y∈U. In this case, $G_{i}^{\prime }$ only contains nodes in U. Property (A) holds trivially. And the relation in property (B) reduces to $G_{i}^{\prime }\left(U\right)=\cup_{k=1}^{n}{\left[G_{i}\left(U\right)\right]}^{k}=G_{i}\left(U\right)$. By the induction hypothesis, we know $G_{i}^{\prime }=G_{i}$ and thus $G_{i+1}^{\prime }=G_{i+1}$. Property (B) holds. This concludes the proof. □

The proof of Lemma A4 requires the following result on eliminating variables from graphs.

**Lemma** **A3.**
*Consider a DAG G, a subset U of its nodes, and a node H in G where H∉U. Let $G_{1}$ be the moral graph of G, and $G_{2}$ be the result of eliminating all nodes other than {H}∪U from $G_{1}$. For any node X∈U, X is adjacent to H in $G_{2}$ if and only if there exists a path between X and H in $G_{1}$ that does not include a node in U∖{X}.*

**Proof.** We first prove the if direction. Suppose there exists such a path $\left(X,\ldots ,Z_{1},Y,Z_{2},\ldots ,H\right)$ in $G_{1}$. Eliminating node *Y* from $G_{1}$ will lead to a path $\left(X,\ldots ,Z_{1},Z_{2},\ldots ,H\right)$. Since nodes in U∖{X} cannot appear along this path, eliminating all nodes other than {H}∪U will lead to the edge (X,H) in $G_{2}$. We next prove the only if direction by contraposition. Suppose there is no path between *X* and *H* in $G_{1}$ that does not include a node in U∖{X}. There are two cases: (1) there is no path between *X* and *H*; (2) every path between *X* and *H* includes at least one node U∈U∖{X}, which has the form (X,…,U,…,H). In the first case, *X* and *H* will be disconnected in $G_{2}$. In the second case, eliminating all nodes other than {H}∪U from such paths will lead to X→U→H, so *X* cannot be directly adjacent to *H* in $G_{2}$. This concludes the proof. □

**Lemma** **A4.**
*Consider an SCM G and a subset U of its roots. Suppose SCM $G^{\prime }$ is obtained from G by adding a root node H as a parent of some nodes Z in G, where Z∩U=∅. Let π be a U-constrained elimination order for G, and let $\pi^{\prime }$ be a U-constrained elimination order of $G^{\prime }$ obtained from π by placing H just before variables U. If π has width w and $\pi^{\prime }$ has width $w^{\prime }$, then $w^{\prime }\leq \max\left(w+1,|U|\right)$. Moreover, if H has a single child in $G^{\prime }$, then $w^{\prime }=w+1$.*

**Proof****.** Let X denote variables other than U in *G*, and let $U^{\prime }=U\cup \left\{H\right\}$. Suppose we first eliminate variables X, then *H*, and finally U from $G^{\prime }$ using order $\pi^{\prime }$. This results in a graph sequence $G_{1}^{\prime },\ldots ,G_{j}^{\prime },G_{j+1/2}^{\prime },G_{j+1}^{\prime },\ldots ,G_{j+k}^{\prime }$ where j=|X| and k=|U|. Here, $G_{j+1/2}^{\prime }$ is obtained by eliminating all variables X from $G_{1}^{\prime }$, and $G_{j+1}^{\prime }$ is obtained by eliminating *H* from $G_{j+1/2}^{\prime }$. We claim:

- if i≤j, then for each node X≠H in $G_{i}^{\prime }$, we have $G_{i}^{\prime }\left(X\right)\subseteq G_{i}\left(X\right)\cup \left\{H\right\}$.
- if i>j, then for each node X≠H in $G_{i}^{\prime }$, we have $G_{i}^{\prime }\left(X\right)\subseteq U^{\prime }$. Moreover, if *H* has a single child in $G^{\prime }$, then $G_{i}^{\prime }\left(X\right)=G_{i}\left(X\right)$.

We first show that the above claim implies the lemma, and then follow by proving the claim. Suppose we are eliminating variable *Y* from $G^{\prime }$. If $Y\notin U^{\prime }$, then i≤j and the above claim implies $C^{\prime }\left(Y\right)\subseteq C\left(Y\right)\cup \left\{H\right\}$. If $Y\in U^{\prime }$ then i>j and the above claim implies $C^{\prime }\left(Y\right)\subseteq U^{\prime }$, and $C^{\prime }\left(Y\right)=C\left(Y\right)$ when *H* has a single child. This guarantees the statement of the lemma: $w^{\prime }\leq \max\left(w+1,|U|\right)$, and $w^{\prime }=w+1$ if *H* has a single child in $G^{\prime }$. We next prove our claim by induction. Let Z denote the children of *H* in $G^{\prime }$. When constructing the moral graph $G_{1}^{\prime }$ from $G^{\prime }$, the introduction of node *H* causes two classes of edges that do not exist in $G_{1}$ to be added to $G_{1}^{\prime }$: (Z,H) for Z∈Z, and (Y,H) if *Y* is a parent of some node Z∈Z, that is *Y* and *H* are common parents of some node *Z*. All of these extra edges are incident on *H*, meaning that for any node *X* in $G_{1}^{\prime }$, $G_{1}^{\prime }\left(X\right)\subseteq G_{1}\left(X\right)\cup \left\{H\right\}$. Thus, our claim holds for $G_{1}^{\prime }$. Next, assume our claim holds for $G_{i}^{\prime }$ (induction hypothesis) and consider $G_{i+1}^{\prime }$. We have two cases. **Case:** i≤j. Let Y=π(i). Consider each node *X* in $G_{i+1}^{\prime }$. If node *X* is not a neighbor of *Y* in $G_{i}/G_{i}^{\prime }$, then *X* is not affected by the elimination of *Y*, i.e., $G_{i+1}^{\prime }\left(X\right)=G_{i}^{\prime }\left(X\right)$ and $G_{i+1}\left(X\right)=G_{i}\left(X\right)$. So the claim holds by the induction hypothesis. Otherwise, we can bound $G_{i+1}^{\prime }\left(X\right)$ as follows:

$$\begin{array}{ccc} G_{i+1}^{\prime }\left(X\right) & =G_{i}^{\prime }\left(X\right)\cup G_{i}^{\prime }\left(Y\right)\setminus \left\{Y\right\} & \mathrm{by}\;\mathrm{the}\;\mathrm{definition}\;\mathrm{of}\;\mathrm{elimination}\\ \; & \subseteq \left(G_{i}\left(X\right)\cup \left\{H\right\}\right)\cup \left(G_{i}\left(Y\right)\cup \left\{H\right\}\right)\setminus \left\{Y\right\} & \;\mathrm{by}\;\mathrm{the}\;\mathrm{induction}\;\mathrm{hypothesis}\\ \; & \subseteq \left(G_{i}\left(X\right)\cup G_{i}\left(Y\right)\setminus \left\{Y\right\}\right)\cup \left\{H\right\}\\ \; & \subseteq G_{i+1}\left(X\right)\cup \left\{H\right\} \end{array}$$

**Case:** i>j. For this case, $G_{i}^{\prime }$ only contains nodes in $U^{\prime }$. It is trivial that $G_{i}^{\prime }\left(X\right)\subseteq U^{\prime }$ for each node *X* in $G_{i}^{\prime }$. Recall that eliminating *H* from $G_{j+1/2}^{\prime }$ results in $G_{j+1}^{\prime }$. By the induction hypothesis, all extra edges in $G_{j+1/2}^{\prime }$ that do not exist in $G_{j+1}$ must be incident on *H*. Consider the special case where *H* has a single child in $G^{\prime }$. We claim that in this case, every two nodes in $G_{j+1/2}^{\prime }\left(H\right)$ are adjacent in $G_{j+1/2}^{\prime }$, meaning that the neighbors of *H* already form a clique in $G_{j+1/2}^{\prime }$. Thus, eliminating *H* from $G_{j+1/2}^{\prime }$ will not add any fill-in edges in $G_{j+1}^{\prime }$. This guarantees $G_{j+1}^{\prime }=G_{j+1}$, i.e, $G_{i}^{\prime }\left(X\right)=G_{i}\left(X\right)$ for all i>=j+1. We finally turn to proving this claim by contradiction. Suppose that node $U_{1}$ and $U_{2}$ are neighbors of *H* in $G_{j+1/2}^{\prime }$ but are not adjacent in $G_{j+1/2}^{\prime }$. By Lemma A3, in $G_{1}^{\prime }$, there must be a path $P_{1}$ between $U_{1}$ and *H* that does not include nodes in $U\setminus \{U_{1}\}$, and a path $P_{2}$ between $U_{2}$ that does not include nodes in $U\setminus \{U_{2}\}$. Since *H* is a root and only has one child *Z* in $G^{\prime }$, $P_{1}$ must have the form $\left(U_{1},\ldots ,Z,H\right)$ in $G_{1}^{\prime }$ and $P_{2}$ must have the form $\left(U_{2},\ldots ,Z,H\right)$ in $G_{1}^{\prime }$. Thus, there must be a path $\left(U_{1},\ldots ,Z,\ldots ,U_{2}\right)$ in $G_{1}^{\prime }$ that does not contain nodes in $U^{\prime }\setminus \left\{U_{1},U_{2}\right\}$. By Lemma A3, after eliminating all nodes other than $U^{\prime }$ from $G_{1}^{\prime }$, $U_{1}$ and $U_{2}$ must be adjacent in $G_{j+1}^{\prime }$. This leads to a contradiction. □

## Appendix J. Proof of Proposition 2

**Proof.** Suppose the decision-AC is evaluated at input $u^{*}$ which is some instantiation of U. Consider any +-node *n* with decision variable *X*. If X∈U, it is easy to verify that at most one child of *n* can be nonzero—the one with indicator $\lambda_{x^{*}}$, where $x^{*}$ is the state of *X* in $u^{*}$. If X∉U, we claim that node *n* cannot depend on U (i.e., vars(n)∩U=∅). Hence, this decision-AC is U-deterministic by Definition 8. We prove this claim by contradiction. Suppose node *n* does depend on some variable U∈U, which means there is an indicator $\lambda_{u}$ below *n*. By condition (2) of Definition 8, this indicator $\lambda_{u}$ must be attached to some +-nodes *m* with decision variable *U*. This implies that *m* is below *n*, which contradicts condition (1). □

## Appendix K. Proof of Theorem 7

**Proof****.** To prove Theorem 7, we need the notion of a subcircuit, which is introduced in [43] and studied extensively in [45]. **Definition** **A1.** *Let* AC(X) *be a decomposable and smooth circuit. A complete subcircuit α of* AC *is obtained by visiting the circuit nodes top-down starting at the root: if a ∗-node is visited, visit all its children, and if a +-node is visited, visit exactly one of its children. The term of α is the set of variable values appearing in indicators of α, and the coefficient of α is the product of all parameters in α.* A complete subcircuit must include exactly one indicator for every variable in X. Hence, the term of each complete subcircuit corresponds to an instantiation x of X, so the subcircuit is called an x-subcircuit. Evaluating AC(x) amounts to summing the coefficients of all x-subcircuits. Furthermore, ref. [45] showed that if the circuit is also deterministic, then for any instantiation x of X, if AC(x)≠0, there is a unique x-subcircuit whose coefficient is nonzero. Denote this subcircuit by $\alpha_{x}$. We know $\alpha_{x}$ has coefficient $p_{1}={\mathrm{Pr}}_{1}\left(x\right)$ under $\Theta_{1}$ and coefficient $p_{2}={\mathrm{Pr}}_{2}\left(x\right)$ under $\Theta_{2}$. By construction, $\alpha_{x}$ must have coefficient $p_{3}=p_{1}/p_{2}$ under $\Theta_{3}$. Hence, $\mathrm{AC}\left(x\right)=p_{3}={\mathrm{Pr}}_{3}\left(x\right)$ under $\Theta_{3}$. This proves Theorem 7 (Without determinism, an AC may have multiple x-subcircuits for a given x, and dividing corresponding parameters may not give the correct result. For a simple example, consider $\mathrm{AC}\left(X\right)=a\cdot \lambda_{x}+b\cdot \lambda_{\bar{x}}+c\cdot \lambda_{x}$. AC is not deterministic; it has two complete subcircuits (x,a) and (x,c) for *x*. We have $\mathrm{AC}\left(x\right)=a_{1}+b_{1}$ under $\Theta_{1}$ and $\mathrm{AC}\left(x\right)=a_{2}+b_{2}$ under $\Theta_{2}$. After dividing corresponding parameters, AC(x) produces $a_{1}/a_{2}+b_{1}/b_{2}$ instead of $\left(a_{1}+b_{1}\right)/\left(a_{2}+b_{2}\right)$). □

## Appendix L. Proof of Lemma 2

**Proof.** By Lemma A3, every two nodes $U_{1}$ and $U_{2}$ in S will be adjacent after all nodes other than U are eliminated from $G^{\prime }$. Thus, nodes in S will form a clique after all nodes other than U are eliminated. This leads to a cluster of size |S| during the elimination process, so |S| is a lower bound for the width of any U-constrained elimination order. □
